# Supplementary material for: Mechanism of K63-linked polyubiquitin recognition and cleavage by the BRCA1-A complex
Source: Nat Commun. 2026 Aug 11;17:8091. doi: 10.1038/s41467-026-75795-y (PMC13462971; doi:10.1038/s41467-026-75795-y)
Supplement: Supplementary file 1 [file 41467_2026_75795_MOESM1_ESM.pdf]

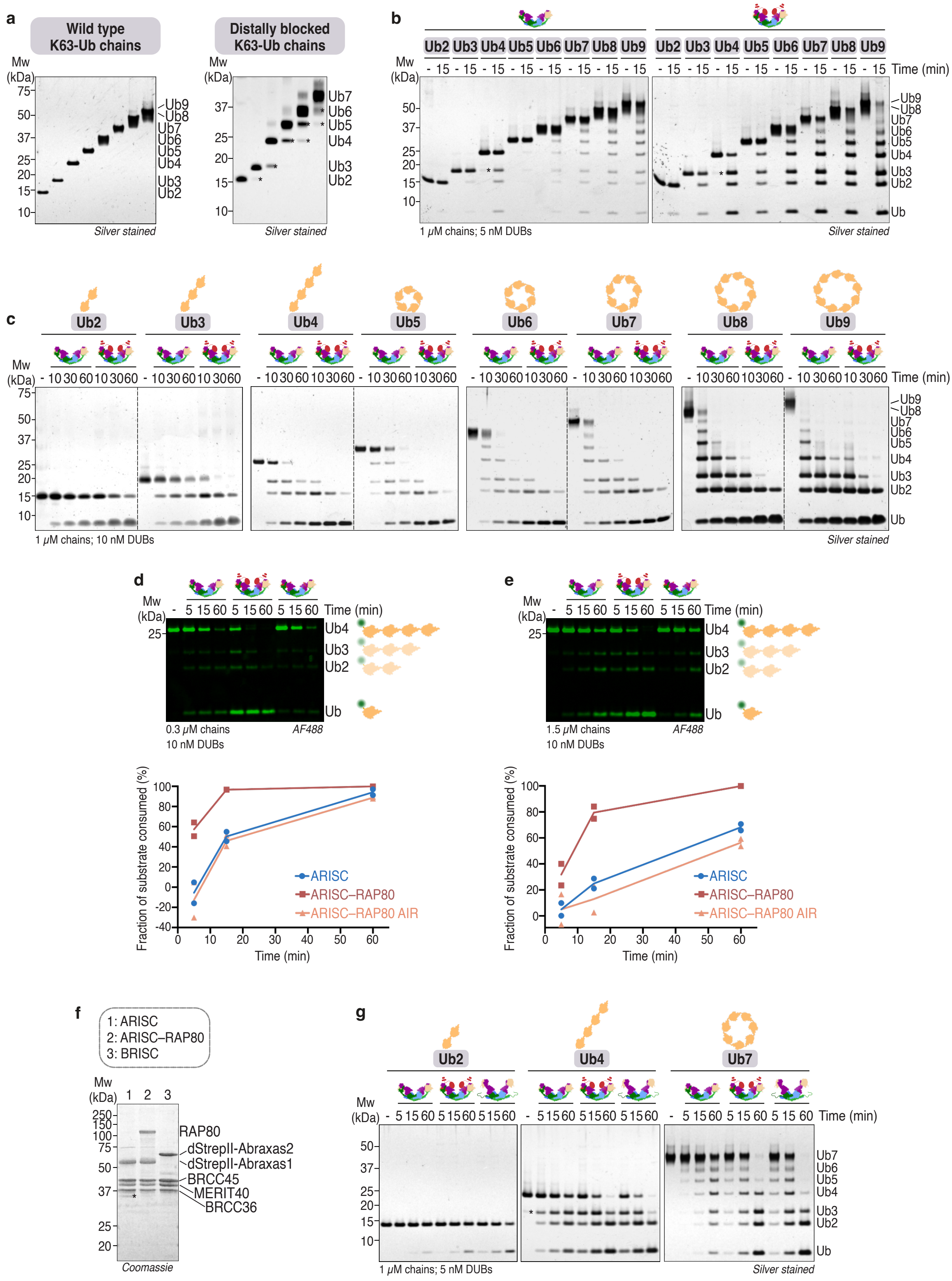

Supplementary Fig. 1: Preparation of protein complexes and ubiquitin chains, and assessment of DUB activities.

a, SDS-PAGE analysis of wild type (WT) and distally blocked K63-linked ubiquitin chains. **b,** Reactions depicted in Fig. 1c (at 15 min time point) were re-loaded side-by-side on the same gels to better highlight differences in substrate cleavage between ARISC and ARISC–RAP80. The final concentration of K63-linked ubiquitin chains was 1 μM, while ARISC and ARISC–RAP80 were

assayed at a final concentration of 5 nM. Cleavage activity was analysed by SDS-PAGE and silver staining. Data are representative of two independent experiments. **c,** K63-linked ubiquitin chains (1 μM) were incubated with ARISC or ARISC–RAP80 (10 nM) for the indicated time points. Cleavage activity was analysed as in b. Data are representative of three independent experiments. **d,e,** Alexa-Fluor 488 (AF488) labelled distally (AF488-^{Cys}Ub4^{K63R}) blocked K63-Ub4 chains (d, 0.3 μM; e, 1.5 μM) were incubated with ARISC, ARISC–RAP80, or ARISC–RAP80 AIR (10 nM) for the indicated time points. Cleavage activity was analysed by SDS-PAGE, and gels were subsequently scanned as described in Fig. 1f (top). The disappearance of the K63-Ub4 parent band was quantified using densitometry, and plotted as fraction of substrate consumed (%). Individual data points from two independent experiments are shown, with a line passing through the mean (bottom). **f,** SDS-PAGE analysis of ARISC, ARISC–RAP80, and BRISC complexes. dStrepII, double StrepII tag. * indicates Abraxas1 degradation product. **g,** K63-Ub2, -Ub4, and -Ub7 chains (1 μM) were incubated with ARISC, ARISC–RAP80, or BRISC (5 nM) for the indicated time points. Cleavage activity was analysed as in b. Data are representative of two independent experiments. * indicates lower molecular weight ubiquitin species. Ub, ubiquitin; DUB, deubiquitylating enzyme. Source data are provided as a Source Data file.

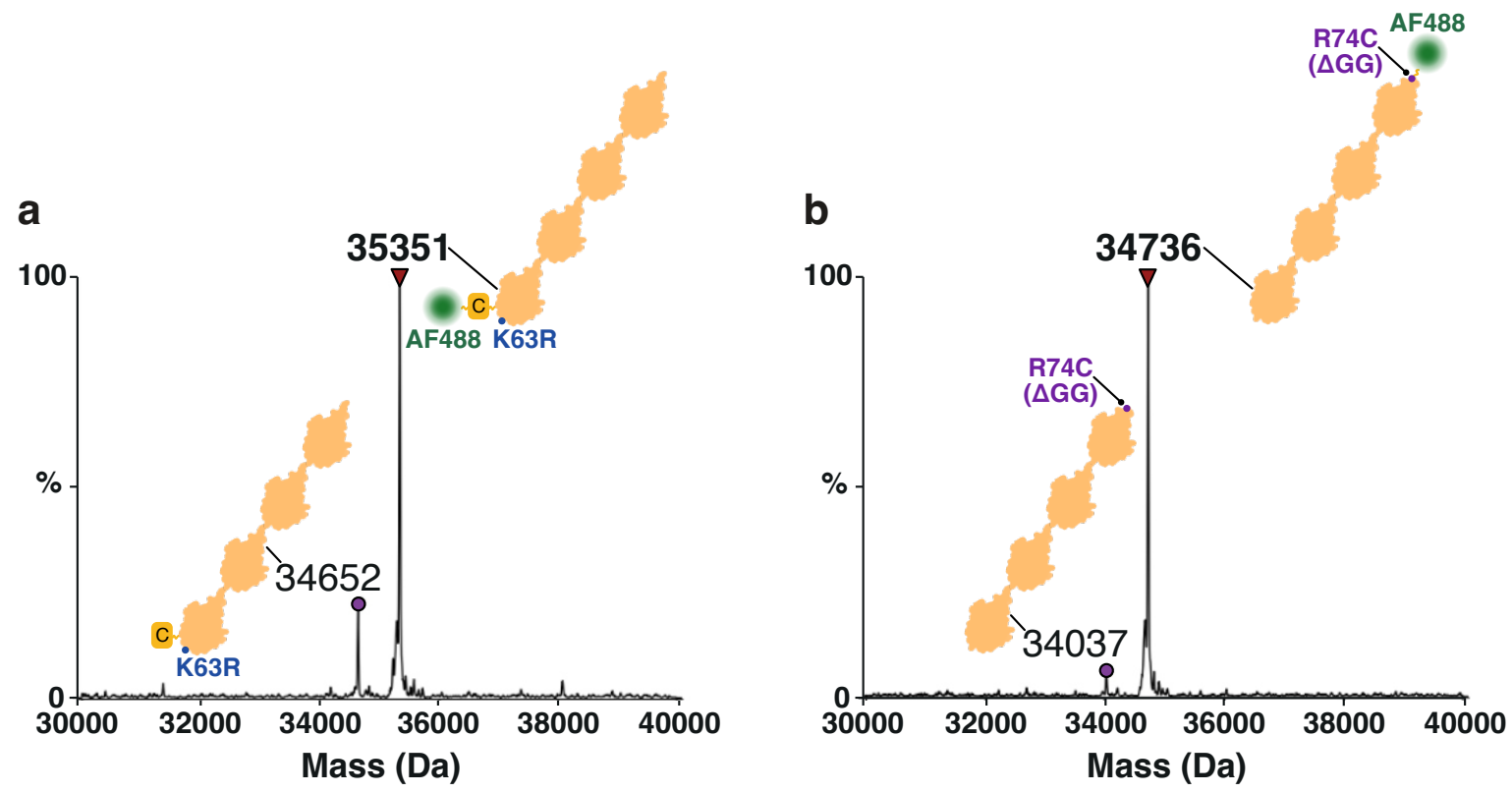

c

AF488 labelled distally (Cys0-ubiquitin K63R) blocked K63-linked tetraubiquitin chain			
**Ubiquitin chains**	**Measured Mw (Da)**	**Calculated Mw (Da)**	**Difference (Da)**
Ub4 (Cys0-Ub K63R)	34652	34652.89	-0.89
AF488-Ub4 (Cys0-Ub K63R)	35351	35350.89	0.11
**AF488 labelled proximally (R74C ΔGG) blocked K63-linked tetraubiquitin chain**			
**Ubiquitin chains**	**Measured Mw (Da)**	**Calculated Mw (Da)**	**Difference (Da)**
Ub4 (R74C ΔGG)	34037	34038.21	-1.21
AF488-Ub4 (R74C ΔGG)	34736	34736.21	-0.21

d

Wild type K63-linked ubiquitin chains			
**Ubiquitin chains**	**Measured Mw (Da)**	**Calculated Mw (Da)**	**Difference (Da)**
Ub2	17111	17111.68	-0.68
Ub3	25658	25658.52	-0.52
Ub4	34205	34205.36	-0.36
Ub5	42734	42752.20	-18.20
Ub6	51280	51299.04	-19.04
Ub7	59827	59845.88	-18.88
Ub8	68375	68392.72	-17.72
Ub9	76922	76939.56	-17.56
**Distally blocked K63-linked ubiquitin chains**			
**Ubiquitin chains**	**Measured Mw (Da)**	**Calculated Mw (Da)**	**Difference (Da)**
Ub2	17626	17627.17	-1.17
Ub3	26173	26174.01	-1.01
Ub4	34720	34720.85	-0.85
Ub5	43267	43267.69	-0.69
Ub6	51815	51814.53	0.47
Ub7	60361	60361.37	-0.37

Supplementary Fig. 2: Validation of Alexa-Fluor 488 labelled distally and proximally blocked K63-linked tetraubiquitin chains by mass spectrometry analyses.

a,b, Intact mass spectra of Alexa-Fluor 488 (AF488) labelled distally (a) and proximally (b) blocked K63-linked Ub4 chains. Observed masses and schematics for the different ubiquitin chains are shown. The positions of the K63R and R74C ΔGG mutants, of the N-terminal cysteine (Cys0), and of the AF488 fluorophore are indicated. Data are representative of a single experiment. **c,** Table summarising the measured and calculated masses from intact mass spectrometry analyses of AF488 labelled distally (AF488-^{Cys}Ub4^{K63R}) and proximally (Ub4$^{R74C\Delta GG}$-AF488) blocked K63-Ub4 chains. Measured and calculated masses for the corresponding unlabelled species are also indicated. **d,** Table summarising the measured and calculated masses from intact mass spectrometry analyses of WT and distally blocked K63-linked ubiquitin chains (see Supplementary Fig. 3). A mass difference of ~ 18 Da in WT Ub5-Ub9 chains indicates the lack of a water molecule.

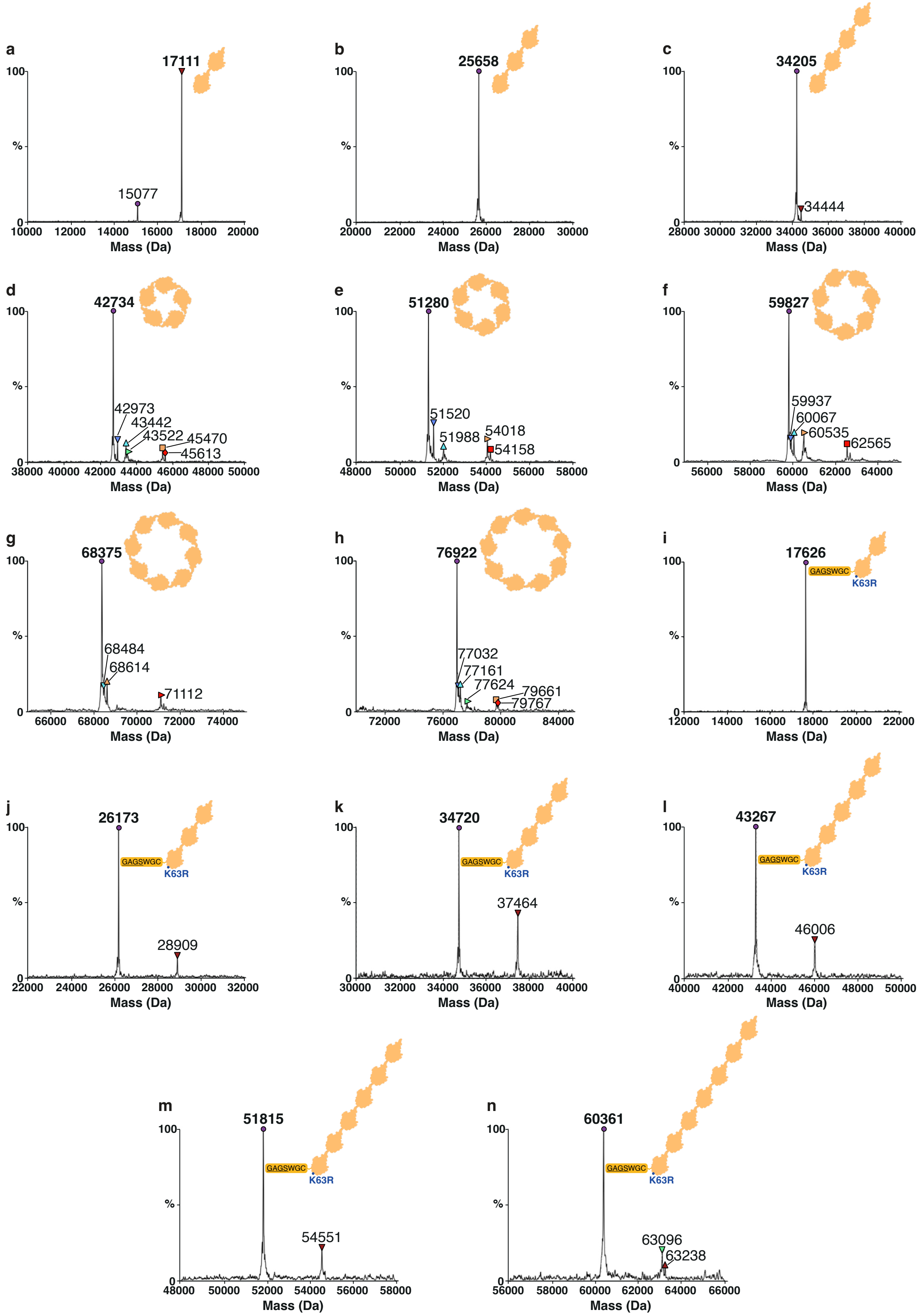

Supplementary Fig. 3: Validation of wild type and distally blocked K63-linked ubiquitin chains by mass spectrometry analyses.

a-h, Intact mass spectrometry analyses of wild type K63-linked Ub2 (a), Ub3 (b), Ub4 (c), Ub5 (d), Ub6 (e), Ub7 (f), Ub8 (g), and Ub9 (h) chains. **i-n,** Intact mass spectra of distally blocked K63-linked

Ub2 (i), Ub3 (j), Ub4 (k), Ub5 (l), Ub6 (m), and Ub7 (n) chains. Observed masses and schematics for the different types and lengths of ubiquitin chains are shown. Amino acids left at the N-terminus of ubiquitin K63R after cleavage of the 8xHis tag, and the position of the K63R mutant, are indicated. Data are representative of a single experiment (see Supplementary Fig. 1a).

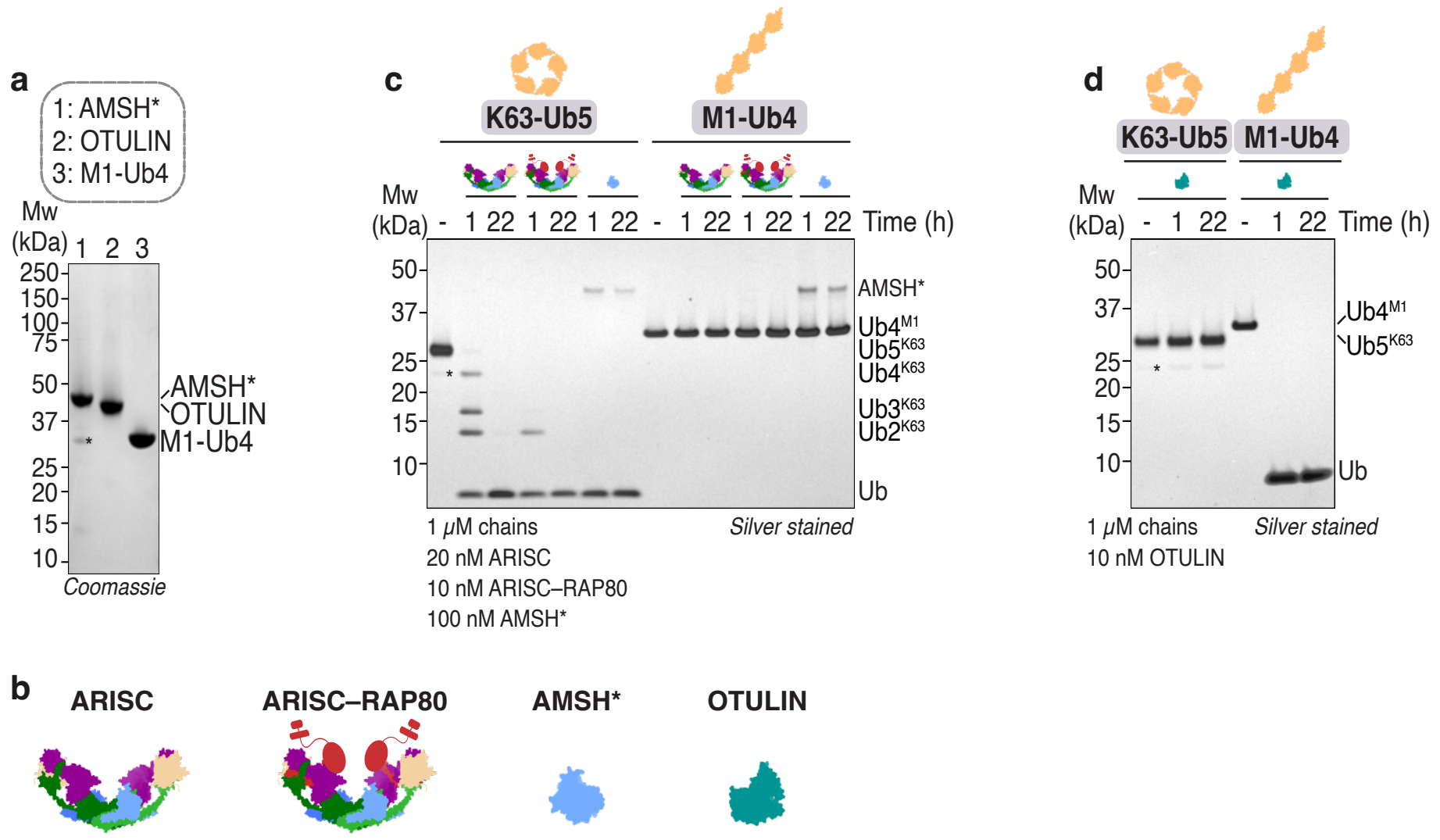

Supplementary Fig. 4: Biochemical analyses of wild type K63-linked pentaubiquitin chains. **a,** SDS-PAGE analysis of AMSH* (a STAM2-AMSH fusion), OTULIN, and M1-linked tetraUb (Ub4) chains. * indicates AMSH* contaminant or degradation product. **b,** Schematics of indicated proteins and protein complexes. **c,d,** Cyclical K63-Ub5 and linear M1-Ub4 chains (1 μM) were incubated with ARISC (20 nM), ARISC–RAP80 (10 nM), and AMSH* (100 nM) (c) or OTULIN (10 nM) (d) for up to 22 h. Cleavage activity was analysed by SDS-PAGE and silver staining. Data are representative of two independent experiments. * indicates lower molecular weight ubiquitin species. Ub, ubiquitin. Source data are provided as a Source Data file.

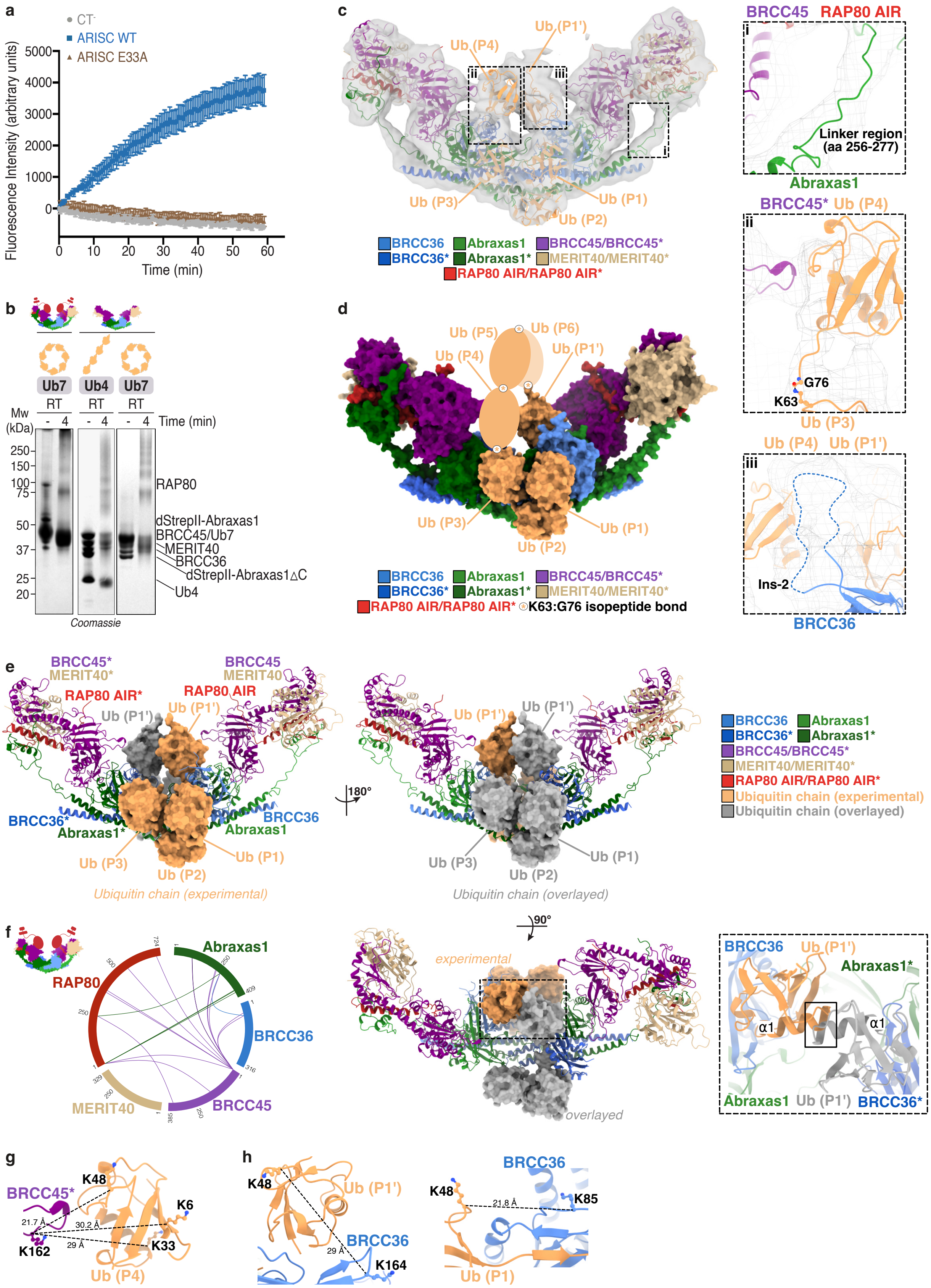

Supplementary Fig. 5: Preparation of ARISC and ARISC–RAP80 complexes for structural studies, and analysis of the ARISC(E33A)–RAP80:K63-Ub7 cryo-EM structure.

a, Deubiquitylase activity of ARISC variants (10 nM) against K63-diUb IQF (50 nM). Data points are mean ± SEM of three independent experiments carried out in technical duplicates. CT$^-$, negative control; WT, wild type. **b,** Glutaraldehyde cross-linking and SDS-PAGE analysis of ARISC(E33A)–

RAP80 or ARISCΔC(E33A) and ubiquitin chains. RT, room temperature; dStrepII, double StrepII tag. **c,** The ARISC(E33A)–RAP80:K63-Ub7 ab-initio map (contour level = 0.105) is depicted in transparent surface. Cartoon models were rigid-body fitted into the density, and colored as in Fig. 1a and Fig. 2a. Dashed black rectangles highlight regions not well resolved in the final composite map (left). Close-up views and structural details of the Abraxas1 linker region (i), putative BRCC45*:Ub (P4) interface (ii), and BRCC36 Ins-2 (iii). The ab-initio map is depicted as mesh; Ub (P3) $K63^{NZ}$ and Ub (P4) $G76^{C}$ are positioned at isopeptide bond distance (1.3 Å), and missing residues are depicted with a dashed line (right). Ins-2, insertion-2. **d,** Surface representation of ARISC(E33A)–RAP80:K63-Ub7, with models colored as in c. Ubiquitin molecules not visualised in the cryo-EM map are depicted as orange ovals. AIR, Abraxas1-interacting region. **e,** The ARISC(E33A)–RAP80:K63-Ub7 structure is shown in three orientations, with a close-up view depicting the BRCC36 and BRCC36* active sites. The BRCC36 molecule and bound ubiquitin chains were overlayed onto the BRCC36* protomer. ARISC–RAP80 subunits and ubiquitin chains are colored as in c; the ubiquitin moieties modelled onto BRCC36* are colored grey. The black rectangle in the close-up view highlights a partial clash between α1 of the two Ub (P1’) molecules. **f,** Chemical cross-linking and mass spectrometry analysis of ARISC(E33A)–RAP80. Proteins and cross-links are depicted as in Fig. 2d-h. Communal cross-links from two independent experiments are shown. **g,h,** Close-up views and structural details of BRCC45*:Ub (P4) (g), and of BRCC36:Ub (P1’) and BRCC36:Ub (P1) (h) interfaces. Structural models are colored as in c. Cross-linked residues are shown as ball & sticks. Distances are indicated with dashed black lines and measured in Ångstrom (Å). Ub, ubiquitin. Source data are provided as a Source Data file.

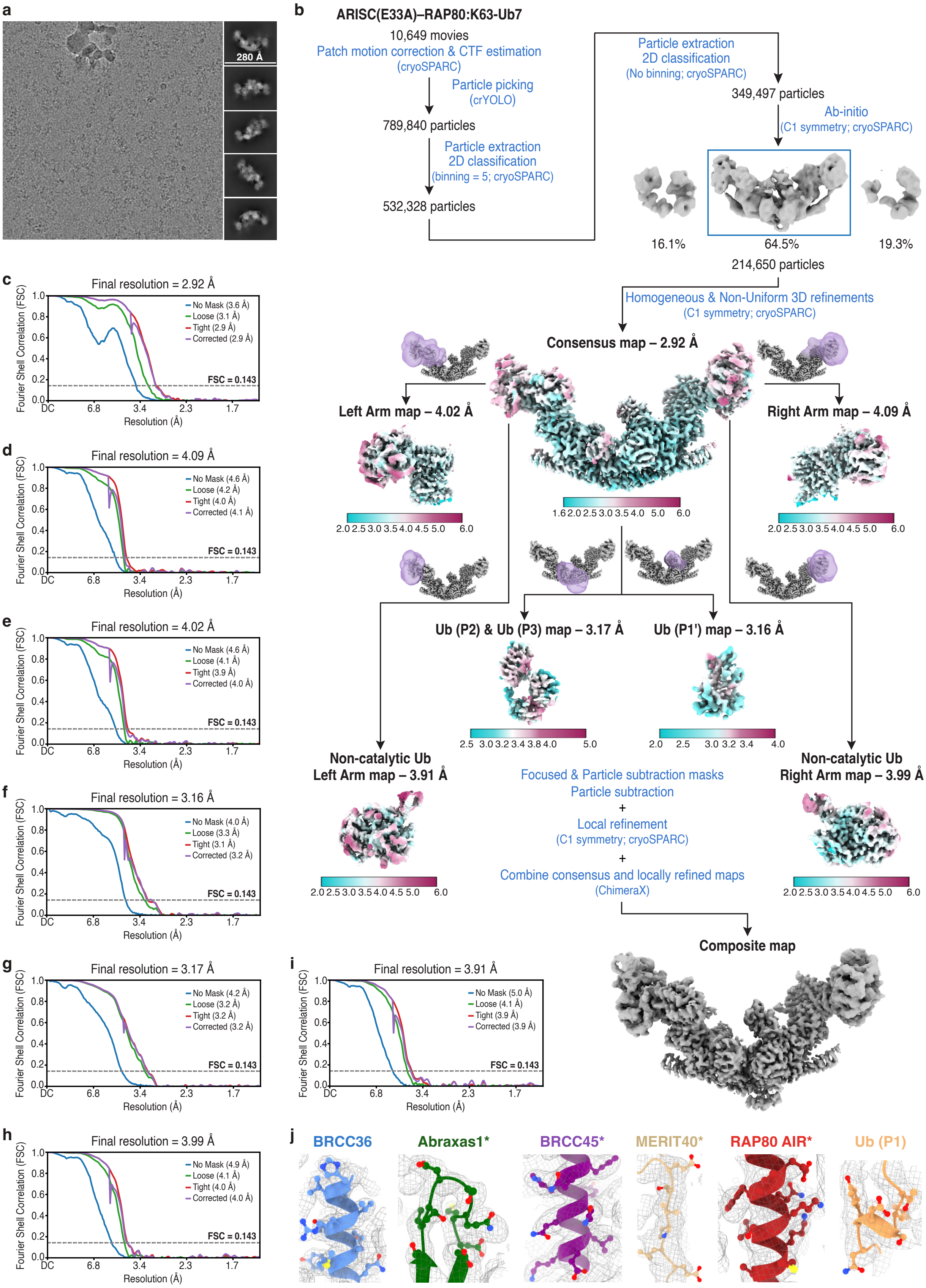

Supplementary Fig. 6: Cryo-EM analysis of the ARISC(E33A)–RAP80:K63-Ub7 complex. **a,** Representative micrograph (out of 10,649 collected movies) and 2D class averages (out of 349,497 selected particles) for the ARISC(E33A)–RAP80:K63-Ub7 dataset. **b,** Flowchart of data processing strategy. The boxed ab-initio model was selected for subsequent processing. The final consensus and locally refined maps are shown and colored by local resolution. See Supplementary

Table 1 for details. **c-i,** Fourier shell correlation (FSC) curves for the final ARISC(E33A)–RAP80:K63-Ub7 consensus map (c), and for the locally refined right (d) and left (e) arm, Ub (P1’) (f), Ub (P2) & Ub (P3) (g), and the non-catalytic ubiquitin right (h) and left (i) arm maps. Resolutions were calculated using the gold-standard FSC cutoff at 0.143 frequency. **j,** Representative density regions (at a contour level of 0.137) from the ARISC(E33A)–RAP80:K63-Ub7 composite map for the different components of the complex. Cartoon models are colored as in Fig. 1a and Fig. 2a, and residues are shown as ball & sticks. AIR, Abraxas1-interacting region; Ub, ubiquitin.

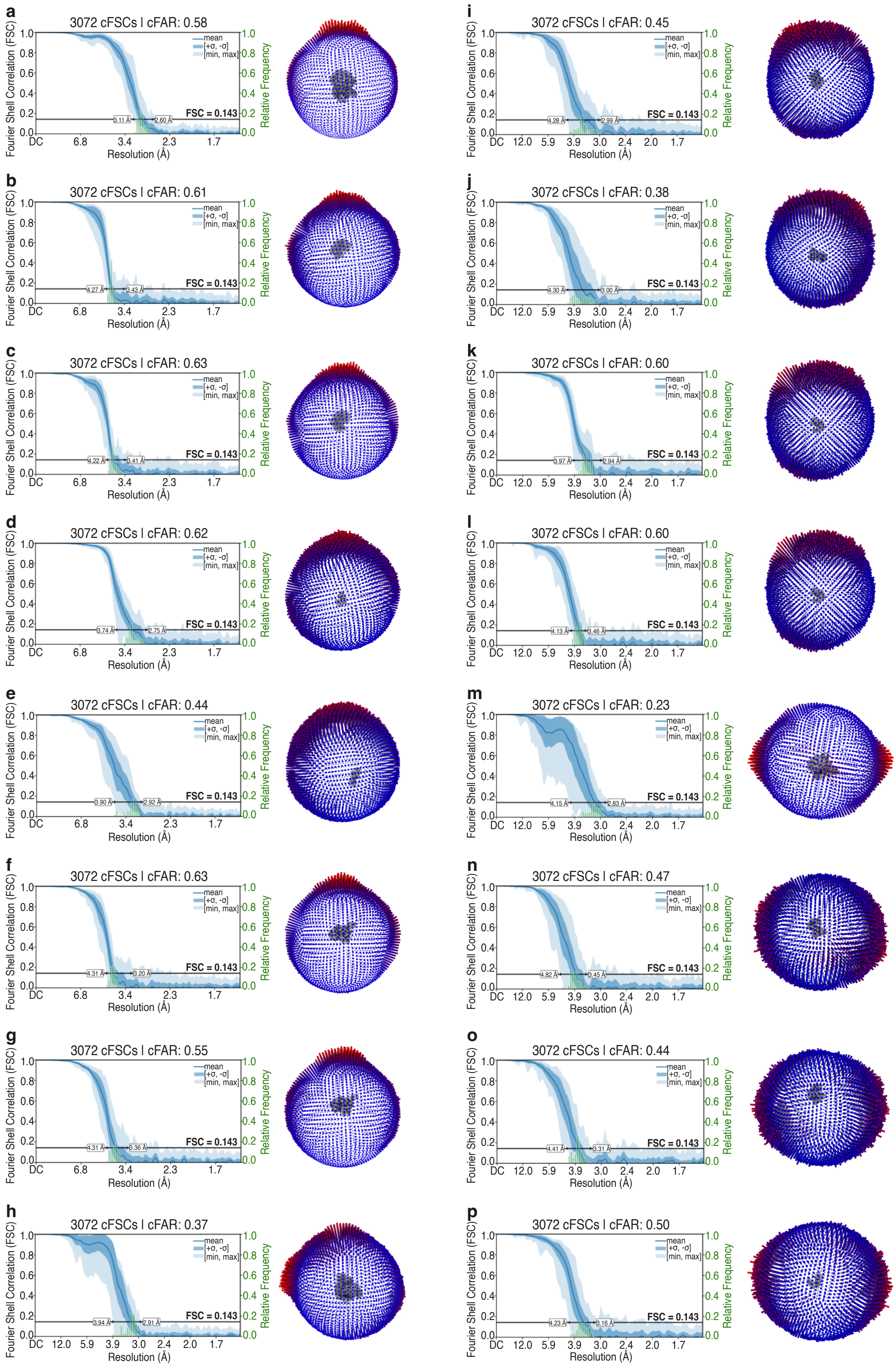

Supplementary Fig. 7: Cryo-electron microscopy validation of the ARISC(E33A)–RAP80:K63-Ub7, ARISCΔC(E33A):K63-Ub7, and ARISCΔC(E33A):K63-Ub4 datasets.

a-g, Plots of conical Fourier shell correlation (FSC) Area Ratio (cFAR) (left) and Euler angle distribution of particles (right) in the ARISC(E33A)–RAP80:K63-Ub7 consensus map (a), and in the locally refined right (b) and left (c) arm, Ub (P1’) (d), Ub (P2) & Ub (P3) (e), and non-catalytic right

(f) and left (g) arm maps (see Supplementary Fig. 6). **h-l,** Plots of cFAR (left) and Euler angle distribution of particles (right) in the ARISCΔC(E33A):K63-Ub7 consensus map (h), and in the locally refined right (i) and left (j) arm, Ub (P1’) (k), and Ub (P2) & Ub (P3) (l) maps (see Supplementary Fig. 12). **m-p**, Plots of cFAR (left) and Euler angle distribution of particles (right) in the ARISCΔC(E33A):K63-Ub4 consensus map (m), and in the locally refined right (n) and left (o) arm, and Ub (P1’) (p) maps (see Supplementary Fig. 13). Rod heights (right) are proportional to the number of particles in each direction. cFAR (left) quantifies the variance of directional half-map Fourier correlations across the viewing sphere ± 1 standard deviation from the mean (area within the light blue lines). Histograms of directional resolutions sampled over the cFAR curves and crossing at the gold-standard FSC cutoff of 0.143 frequency (green bars) are also indicated. cFAR values equal to or higher than 0.5 indicate no significant preferred particle orientation.

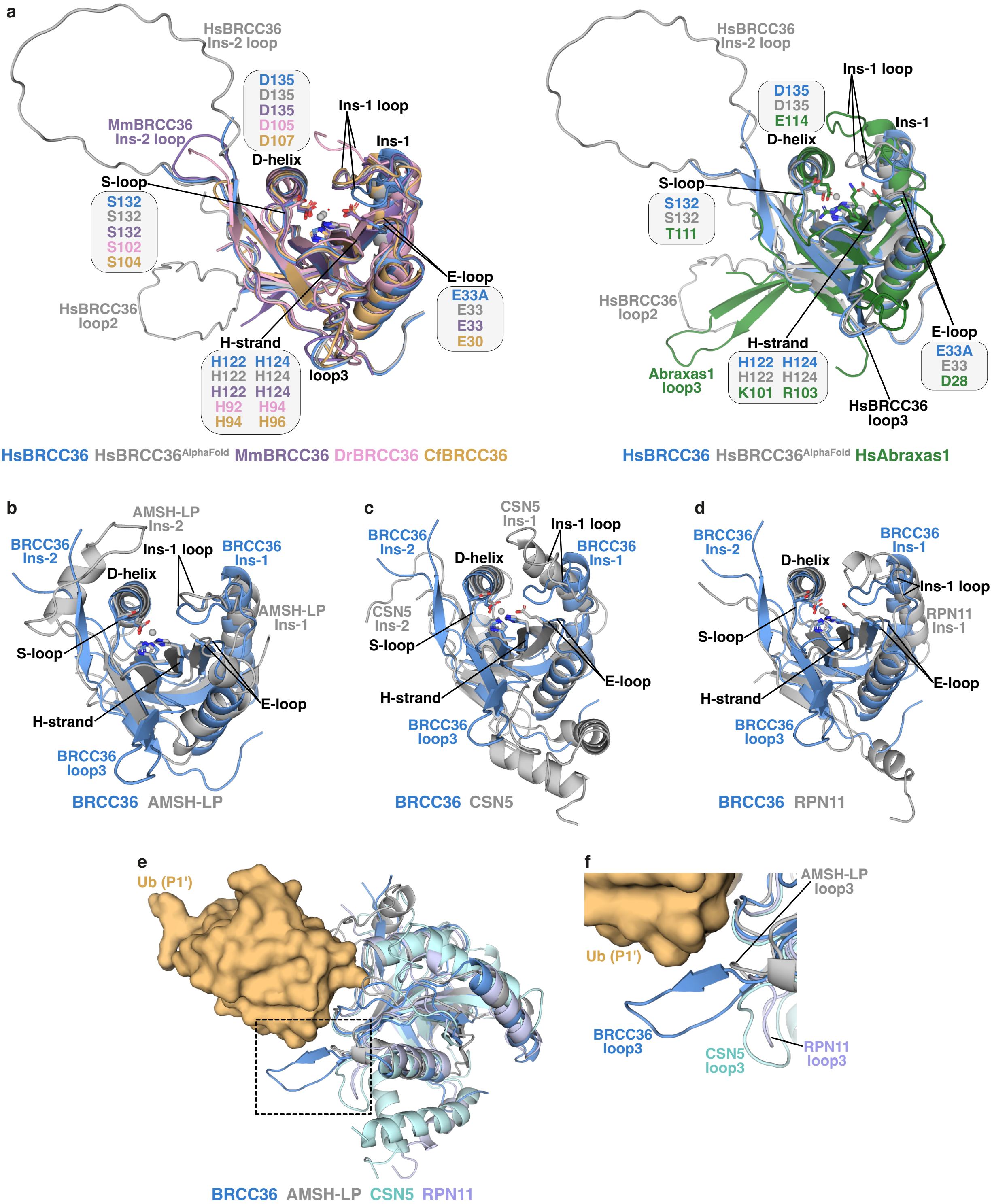

Supplementary Fig. 8: Structural analysis of BRCC36 and Abraxas1 subunits.

a, Superimposition of the BRCC36 MPN domain from human (Hs; from the ARISC(E33A)–RAP80:K63-Ub7 complex and AlphaFold 3 model), mouse (Mm; PDBid = 6GVW), zebrafish (Dr; PDBid = 5CW6), and ant (Cf; PDBid = 5CW3) (left) or between the human BRCC36 MPN domain (Hs; from the ARISC(E33A)–RAP80:K63-Ub7 complex and AlphaFold 3 model) and the corresponding region of human Abraxas1 (Hs; from the ARISC(E33A)–RAP80:K63-Ub7 complex) (right). Structures are shown as cartoon and colored as indicated; active site residues are labelled and shown as sticks. Representative Zn^{2+} atoms obtained from the human (left and right), zebrafish, and ant (left) structures are depicted as grey spheres; the catalytic water molecule obtained from the mouse structure (left) is shown as a red sphere. Secondary structural elements are indicated. **b-d,**

Overlays between the BRCC36 MPN domain (from the ARISC(E33A)–RAP80:K63-Ub7 complex) and the corresponding regions of AMSH-LP (PDBid = 2ZNV; RMSD = 2.4 Å) (b), CSN5 (PDBid = 4F7O; RMSD = 1.7 Å) (c), and RPN11 (PDBid = 4O8X; RMSD = 2.0 Å) (d). Structures are shown as cartoon and colored as indicated; active site residues are shown as sticks. Representative Zn^{2+} atoms obtained from the ARISC(E33A)–RAP80:K63-Ub7 (b-d) and RPN11 (d) structures are depicted as grey spheres. Secondary structural elements are indicated. Ins, insertion. **e,** Superimposition of the BRCC36 (from the ARISC(E33A)–RAP80:K63-Ub7 complex), AMSH-LP (PDBid = 2ZNV), CSN5 (PDBid = 4F7O), and RPN11 (PDBid = 4O8X) structures. Models are shown as cartoon and colored as indicated; the Ub (P1') molecule (obtained from the ARISC(E33A)–RAP80:K63-Ub7 complex) is shown as surface and colored as in Fig. 2a. A dashed black rectangle highlights the position of loop3 relative to Ub (P1'). **f,** Close-up view of loop3 in the BRCC36, AMSH-LP, CSN5, and RPN11 structures shown in e. Models are depicted and colored as in e. Ub, ubiquitin.

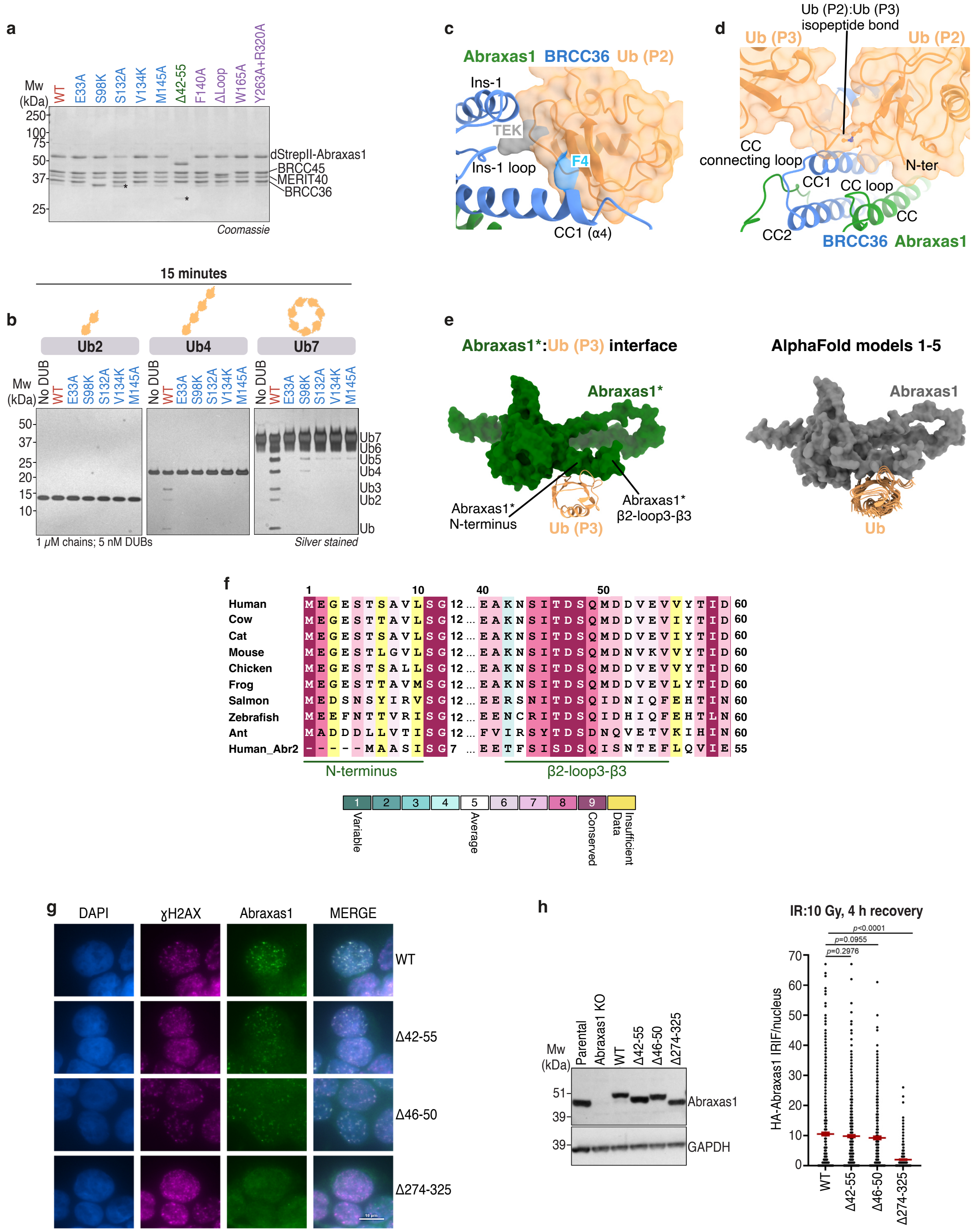

Supplementary Fig. 9: Structural, biochemical, and cell-based analyses and validation of the BRCC36 and Abraxas1 ubiquitin binding sites.

a, SDS-PAGE analysis of ARISC WT (red) and variants bearing mutations or deletions in BRCC36 (blue), Abraxas1 (green), or BRCC45 (purple). BRCC45 ΔLoop refers to deletion of residues 161-170. dStrepII, double StrepII tag. * indicates Abraxas1 degradation product. **b,** K63-Ub2, -Ub4, and -Ub7 chains (1 μM) were incubated with ARISC variants (5 nM) for 15 minutes. Cleavage activity was analysed by SDS-PAGE and silver staining. Data are representative of two independent experiments. DUB, deubiquitylating enzyme. **c,** Close-up view of the BRCC36:Ub (P2) interface. Models are shown as cartoon, and colored as in Fig. 1a and Fig. 2a. The F4 patch and TEK box are

indicated. Ins, insertion. **d,** Close-up view of the region near the Ub (P2):Ub (P3) isopeptide bond. Models are colored as in c. N-ter, N-terminus; CC, coiled coil. **e,** Structural comparisons between the Abraxas1*:Ub (P3) interface (from ARISC(E33A)–RAP80:K63-Ub7; green and orange) and the corresponding AlphaFold 3 models (grey and orange). Abraxas1 and ubiquitin are shown as surface and cartoon, and colored as in c. Ub, ubiquitin. **f,** Multiple sequence alignment of the Abraxas1 N-terminal and β2-loop3-β3 regions across nine species, with the corresponding residues in human Abraxas2 included. Sequence conservation was generated by the Consurf web server[1], and indicated by a score between 1 and 9. The numbers at the top of the alignment refer to human Abraxas1. **g,** Representative images of Abraxas1 IRIF in HT-29 cells expressing WT or mutant proteins 4 h post irradiation (10 Gy). Scale bar is 10 μm. **h,** Western blots showing Abraxas1 protein levels in HT-29 cells reconstituted with WT or mutant proteins (left). Scatter plot showing quantification of the data described in g. Data represent mean ± SEM from at least 350 nuclei (n = 2 independent experiments); *p* values were calculated with unpaired two-tailed t test (right). KO, knockout; WT, wild type. Source data are provided as a Source Data file.

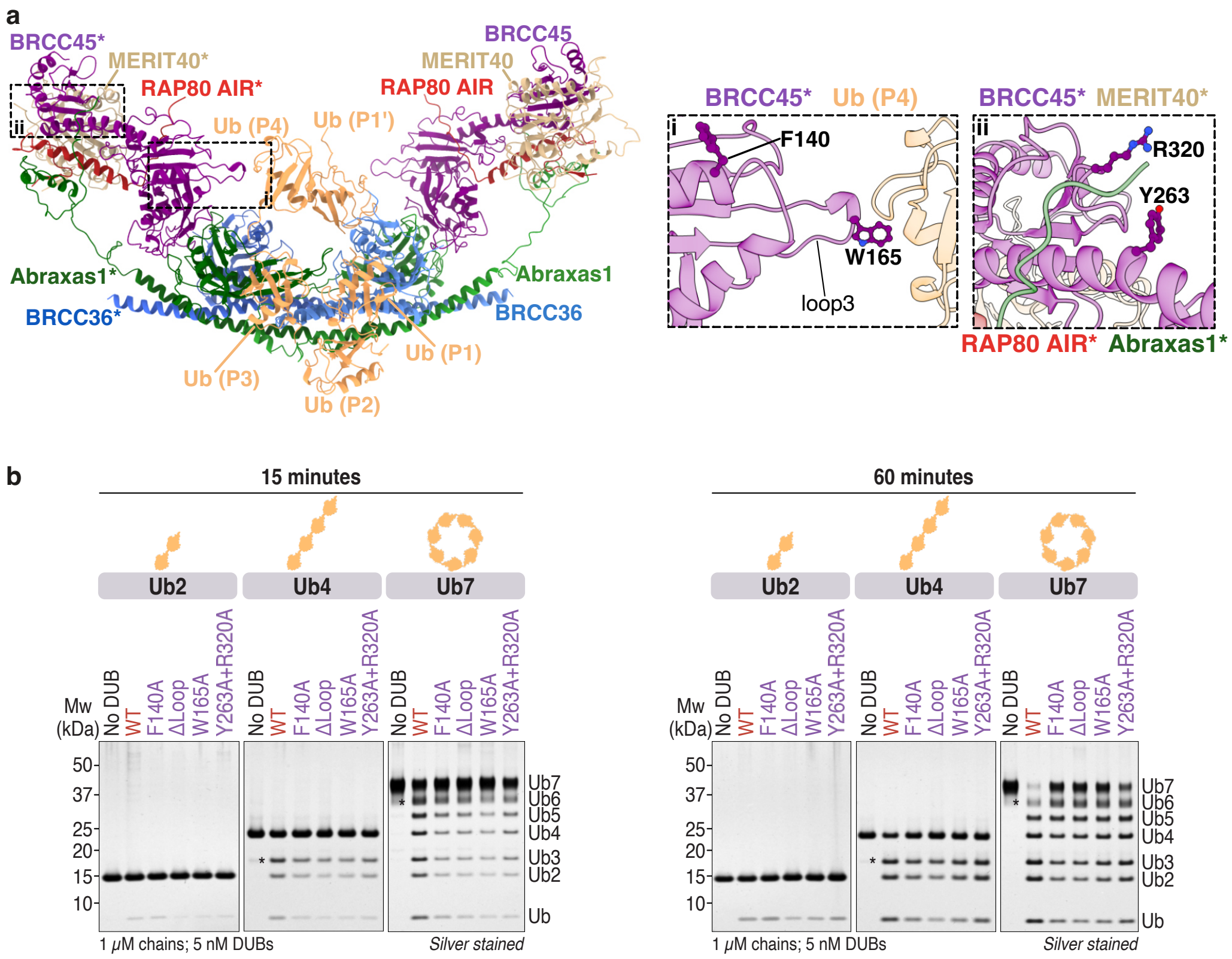

Supplementary Fig. 10: Determining the DUB activity of structure-guided BRCC45 mutants. **a,** The same model of the ARISC(E33A)–RAP80:K63-Ub7 complex shown in Supplementary Fig. 5c is depicted as cartoon representation, with ARISC–RAP80 subunits and ubiquitin chains colored as in Fig. 1a and Fig. 2a. Dashed black rectangles highlight regions analysed by mutagenesis (left). Close-up views and structural details of the predicted interacting residues at the putative BRCC45*:Ub (P4) (i) and BRCC45*:Abraxas1* (ii) interfaces. Residues are shown as ball & sticks (right)**.** AIR, Abraxas1-interacting region. **b,** K63-Ub2, -Ub4, and -Ub7 chains (1 μM) were incubated with ARISC WT or the indicated ARISC variants (5 nM) for up to 60 minutes. Cleavage activity was analysed by SDS-PAGE and silver staining. Data are representative of two independent experiments. BRCC45 ΔLoop refers to deletion of amino acids 161-170. DUB, deubiquitylating enzyme; WT, wild type; Ub, ubiquitin. Source data are provided as a Source Data file.

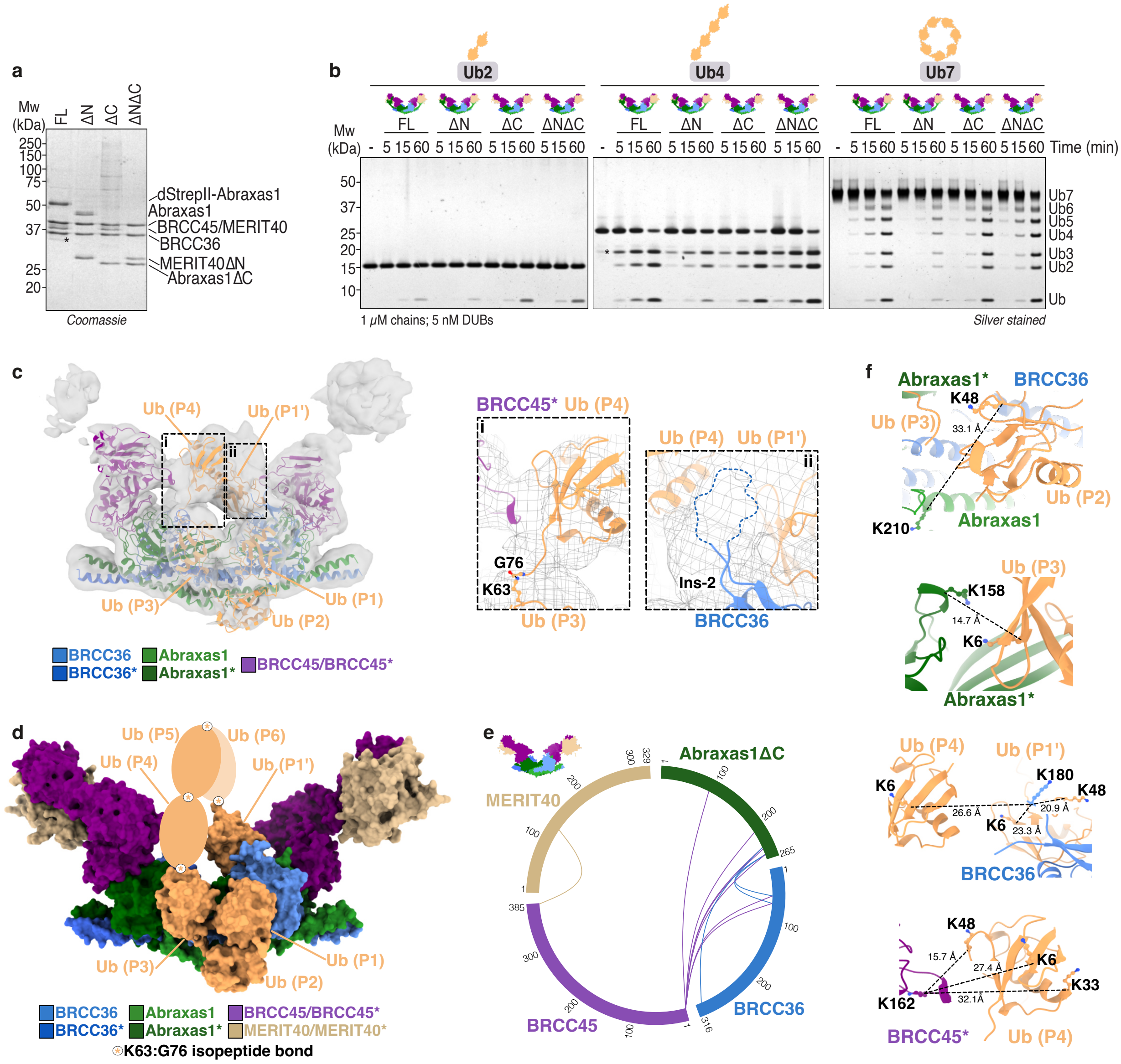

Supplementary Fig. 11: Biochemical analyses of ARISC variants, and validation of the ARISCΔC(E33A):K63-Ub7 and ARISCΔC(E33A):K63-Ub4 cryo-EM structures.
a, SDS-PAGE analysis of FL ARISC, ARISCΔN, ARISCΔC, and ARISCΔNΔC. dStrepII, double StrepII tag. * indicates Abraxas1 degradation product. **b,** K63-Ub2, -Ub4, and -Ub7 chains (1 μM) were incubated with FL ARISC, ARISCΔN, ARISCΔC, or ARISCΔNΔC (5 nM) for the indicated time points. Cleavage activity was analysed by SDS-PAGE and silver staining. Data are representative of two independent experiments. FL, full-length; DUB, deubiquitylating enzyme. **c,** The ARISCΔN(E33A):K63-Ub7 heterogeneous refinement map (contour level = 0.118) is depicted in transparent surface. Cartoon models were rigid-body fitted into the cryo-EM density, and colored as in Fig. 1a and Fig. 2a. Dashed black rectangles highlight regions not well resolved in the final composite map (left). Close-up views and structural details of the putative BRCC45*:Ub (P4) (i) and BRCC36 Ins-2 (ii) interfaces. The cryo-EM heterogeneous refinement map is depicted as mesh; Ub (P3) K63^{NZ} and Ub (P4) G76^{C} are positioned at isopeptide bond distance (1.5 Å), and missing residues are depicted with a dashed line (right). Ins-2, insertion-2. **d,** The ARISCΔC(E33A):K63-Ub7 structure is shown as surface representation, with structural models colored as in **c**. Ubiquitin molecules not visualised in the cryo-EM map are depicted as orange ovals. Owing to low resolution in the extended arm regions, overlayed BRCC45 UEV-C and MERIT40 models are shown for size comparison only. **e,** Chemical cross-linking and mass spectrometry analyses of ARISCΔC. Proteins and cross-links are labelled and coloured as in Fig. 5f-i. Communal cross-links obtained from two

independent experiments are shown. **f,** Close-up views and structural details of the Abraxas1:Ub (P2), Abraxas1*:Ub (P3), BRCC36:Ub (P1'):Ub (P4), and putative BRCC45*:Ub (P4) interfaces. Structural models are colored as in c. Residues involved in cross-links are shown as ball & sticks. Distances are indicated with dashed black lines and measured in Ångstrom (Å). Ub, ubiquitin. Source data are provided as a Source Data file.

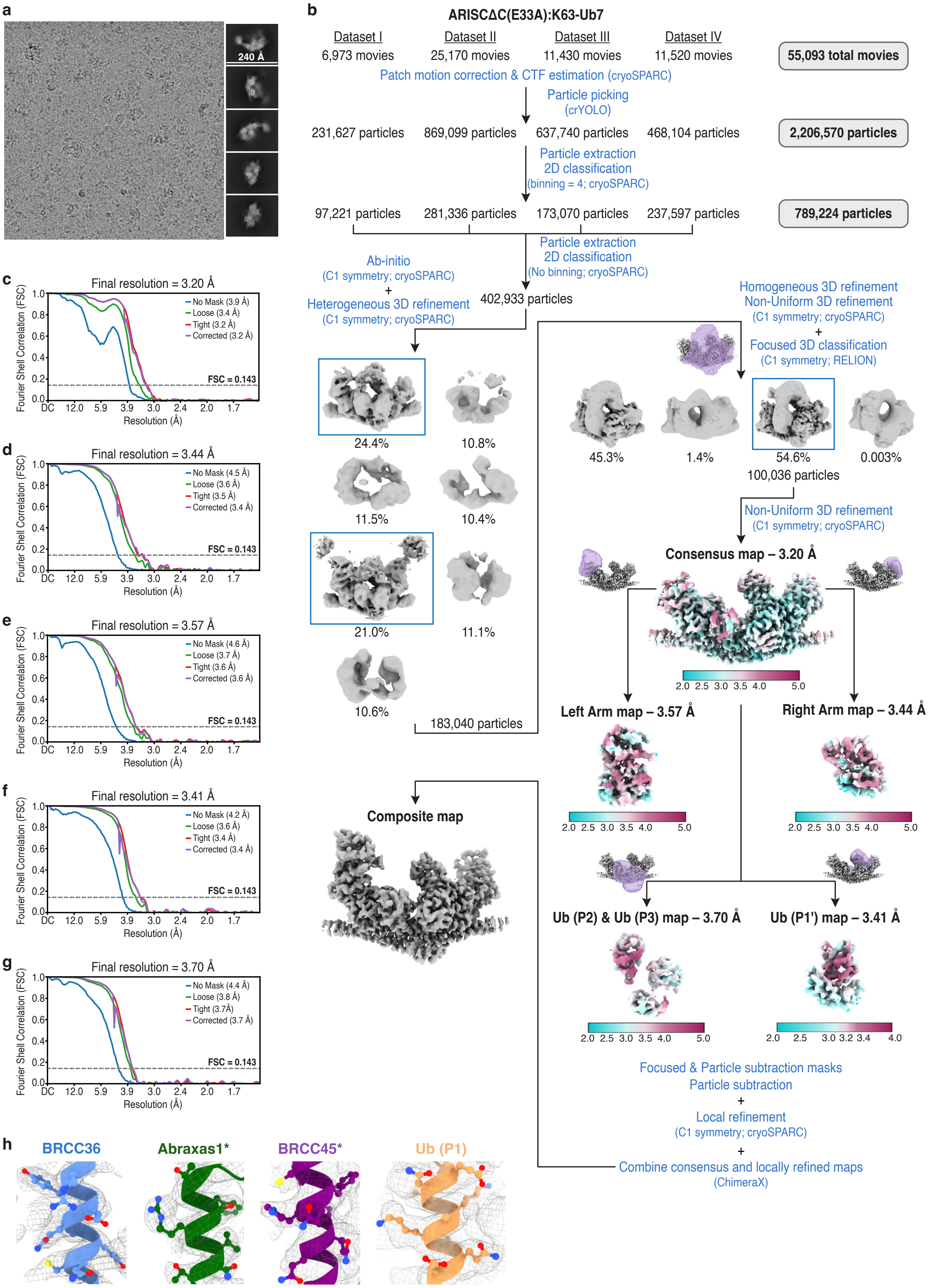

Supplementary Fig. 12: Cryo-EM analysis of the ARISCΔC(E33A):K63-Ub7 complex. **a,** Representative micrograph (out of 55,093 collected movies) and 2D class averages (out of 402,933 selected particles) for the ARISCΔC(E33A):K63-Ub7 dataset. **b,** Flowchart of data processing strategy. Boxed 3D classes were selected for subsequent processing. The final consensus and locally refined maps are shown and colored by local resolution. See Supplementary

Table 1 for details. **c-g,** Fourier shell correlation (FSC) curves for the final ARISCΔC(E33A):K63-Ub7 consensus map (c), and for the locally refined right (d) and left (e) arm, Ub (P1') (f), and Ub (P2) & Ub (P3) (g) maps. Resolutions were calculated using the gold-standard FSC cutoff at 0.143 frequency. **h,** Representative density regions (at a contour level of 0.0514) from the ARISCΔC(E33A):K63-Ub7 composite map for the different components of the complex. Cartoon models are colored as in Fig. 1a and Fig. 2a, and residues are shown as ball & sticks. Ub, ubiquitin.

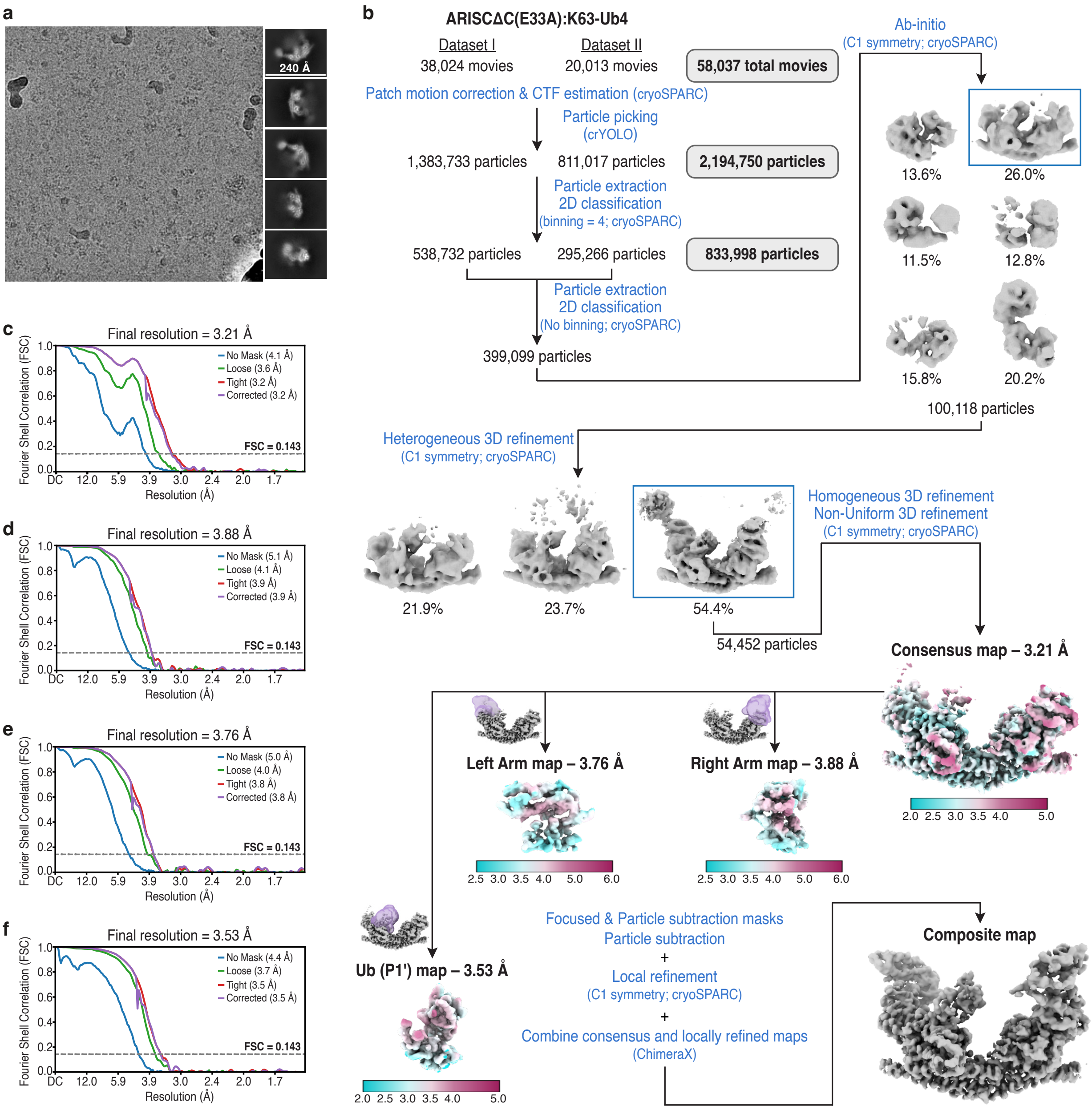

Supplementary Fig. 13: Cryo-EM analysis of the ARISCΔC(E33A):K63-Ub4 complex.
a, Representative micrograph (out of 58,037 collected movies) and 2D class averages (out of 399,099 selected particles) for the ARISCΔC(E33A):K63-Ub4 dataset. **b,** Flowchart of data processing strategy. Boxed ab-initio and 3D classes were selected for subsequent processing. The final consensus and locally refined maps are shown and colored by local resolution. See Supplementary Table 1 for details. **c-f,** Fourier shell correlation (FSC) curves for the final ARISCΔC(E33A):K63-Ub4 consensus map (c), and for the locally refined right (d) and left (e) arm, and Ub (P1') (f) maps. Resolutions were calculated using the gold-standard FSC cutoff at 0.143 frequency.

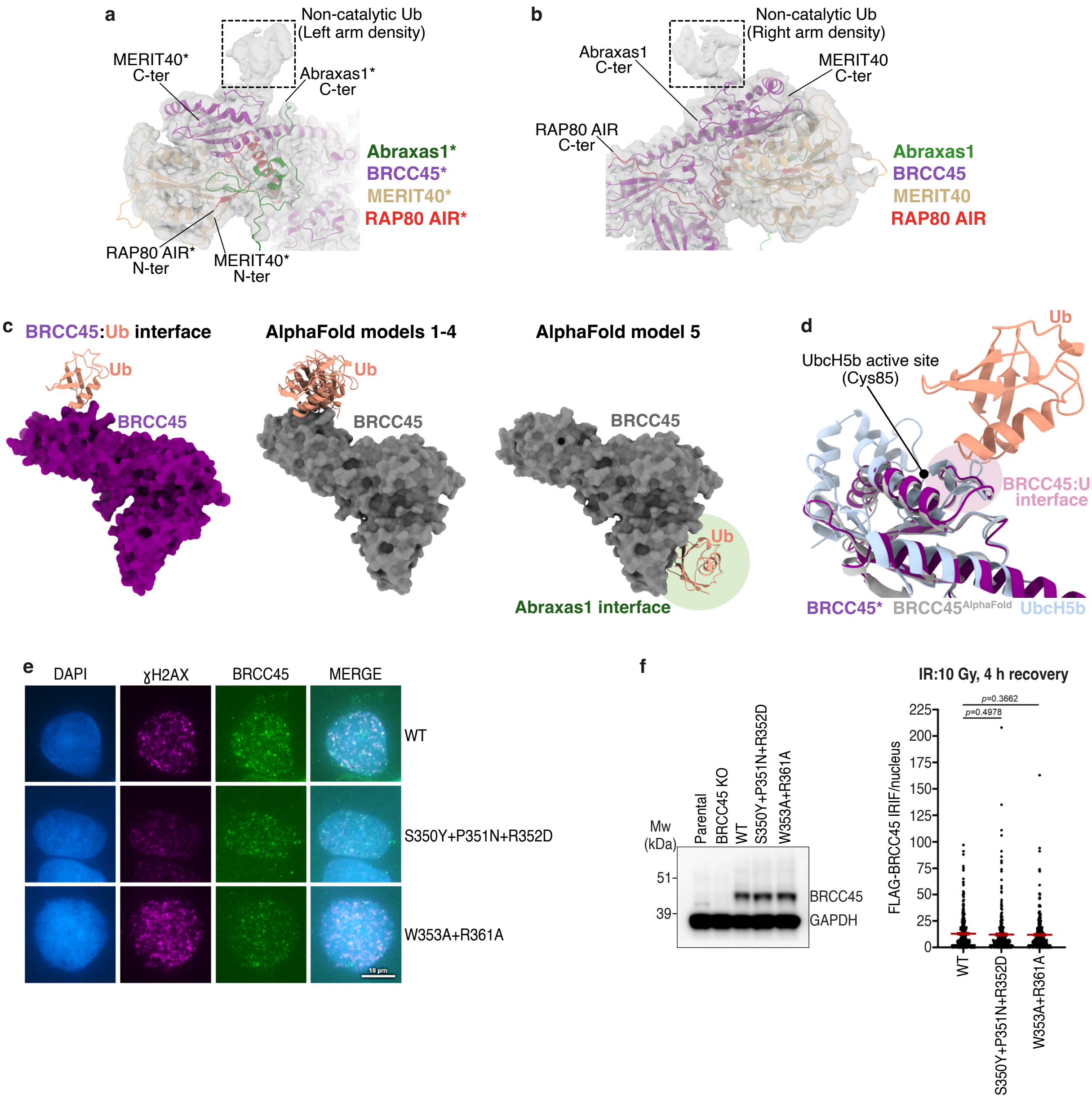

Supplementary Fig. 14: Structural analyses and validation of the ARISC–RAP80 non-catalytic ubiquitin binding sites.

a,b, Close-up views of the left (a) and right (b) ARISC–RAP80 arms from the cryo-EM composite map of the ARISC(E33A)–RAP80:K63-Ub7 complex. The cryo-EM map at contour level of 0.0247 is depicted in transparent surface; cartoon models of ARISC–RAP80 subunits were rigid-body fitted into the cryo-EM density and colored as indicated. Dashed black rectangles highlight the non-catalytic ubiquitin sites located at top of each BRCC45* UEV-C domain (see Fig. 7b). AIR, Abraxas1-interacting region; N-ter, N-terminus; C-ter, C-terminus. **c,** Structural comparisons between the non-catalytic BRCC45*:Ub interface as seen in the ARISC(E33A)–RAP80:K63-Ub7 structure (purple and salmon) and the corresponding interfaces predicted in the AlphaFold 3 models (grey and salmon). BRCC45 and ubiquitin models are shown as surface and cartoon representations, and colored as in Fig. 1a and Fig. 7a. **d,** Structural overlays between the non-catalytic BRCC45*:Ub interface as seen in the ARISC(E33A)–RAP80:K63-Ub7 structure (purple and salmon) and the corresponding BRCC45 region predicted in AlphaFold 3 model 1 (light grey) as well as UbcH5b (PDBid = 3A33) (light blue). The UbcH5b active site cysteine residue (Cys85) and the BRCC45 or UbcH5b regions at the interface with ubiquitin are indicated. Structural models are shown as cartoon; the ubiquitin moiety loaded onto UbcH5b was omitted for clarity. Ub, ubiquitin. **e,** Representative

immunofluorescence images of FLAG-BRCC45 IRIF in HEK293T cells expressing WT or indicated BRCC45 mutants. Cells were irradiated with 10 Gy and recovered for 4 h before fixation. Scale bar is 10 μm. **f,** Western blots showing expression of FLAG-BRCC45 in BRCC45 KO HEK293T cells transfected with WT or mutant BRCC45 expressing plasmids. Cells were harvested 48 h post transfection. GAPDH serves as loading control (left). Scatter plot showing quantification of the data described in e. Data represent mean ± SEM from at least 336 nuclei (n = 2 independent experiments); *p* values were calculated with unpaired two-tailed t test (right). KO, knockout; WT, wild type. Source data are provided as a Source Data file.

Supplementary Table 1: Cryo-EM data collection, refinement and validation statistics

	ARISC(E33A)–RAP80:K63-Ub7<br>EMD-55122<br>PDBid: 9SQY	ARISCΔC(E33A):K63-Ub7<br>EMD-55119<br>PDBid: 9SQW	ARISCΔC(E33A):K63-Ub4<br>EMD-55118<br>PDBid: 9SQV
**Data Collection**			
Microscope		FEI Titan KRIOS	
Detector		Falcon 4i	
Voltage (keV)		300	
Mode		Counting	
Energy Filter (eV)		10	
Pixel size (Å)		0.74	
Magnification (x)		165,000	
Electron dose ($e^-/Å^2$)	45.5	45.4-45.5	45.6-45.9
Exposure (s)	3.4	3.36-3.75	3.4
Electron dose per frame ($e^-/Å^2$)	1.17	1.06-1.17	1.17-1.18
Fractions	39	39-43	39
Total no. of movies	10,649	55,093	58,037
Defocus range (µm)		-0.9 to -3.0	
AFIS range (µm)		10	
**Data Processing**			
Symmetry Point Group		C1	
Initial particle number	789,840	2,206,570	2,194,750
Final particle number	214,650	100,036	54,452
Map resolution (Å)			
Consensus	2.92	3.20	3.21
Right/Left Arms	4.09/4.02	3.44/3.57	3.88/3.76
Ub (P1’)	3.16	3.41	3.53
Ub (P2) & Ub (P3)	3.17	3.70	-
Non-catalytic Ub Right/Left Arms	3.99/3.91		-
FSC threshold		0.143	
Map resolution range (Å)			
Consensus	1.6-6.0	2.0-5.0	2.0-5.0
Right/Left Arms	2.0-6.0/2.0-6.0	2.0-5.0/2.0-5.0	2.5-6.0/2.5-6.0
Ub (P1’)	2.0-4.0	2.0-4.0	2.0-5.0
Ub (P2) & Ub (P3)	2.5-5.0	2.5-5.0	-
Non-catalytic Ub Right/Left Arms	2.0-6.0/2.0-6.0		-
**Refinement**			
Model resolution (Å)	1.8	1.9	1.9
FSC threshold		0.143	
Model composition			
Chains	17	11	9
Non-hydrogen atoms	23,659	13,761	12,545
Protein residues	2,956	1,722	1,572
Ligands	Zn: 2	Zn: 2	Zn: 2
B factors ($Å^2$)			
Protein (min/max/mean)	19.92/264.02/94.61	32.53/201.57/91.13	23.73/174.89/85.20
Ligands (min/max/mean)	84.40/91.04/87.72	58.95/105.71/82.33	62.47/131.97/97.22
Map:model CC			
CC (mask/box/peaks/volume)	0.72/0.77/0.73/0.75	0.75/0.75/0.74/0.77	0.72/0.75/0.72/0.74
Mean CC for ligands	0.75	0.80	0.76
R.m.s. deviations			
Bond lengths (Å)	0.003	0.003	0.002
Bond angles (°)	0.641	0.484	0.507
**Validation**			
MolProbity score	1.79	1.92	2.05
Clash score	9.06	12.9	18.01
Rotamers outliers (%)	0.53	0.19	0.42
Cß outliers (%)	0	0	0
CaBLAM outliers (%)	2.60	2.40	2.50
Ramachandran plot			
Favored (%)	95.64	95.73	95.71
Allowed (%)	4.26	4.27	4.29
Outliers (%)	0.10	0	0

Supplementary Table 2: List of expression constructs

ARISC & BRISC variants

Expression Construct	Species	Cloning/Gene Synthesis	Protein/Sub-complex 1 (name)	Protein/Sub-complex 1 (aa)	Tag	Protein/Sub-complex 2 (name)	Protein/Sub-complex 2 (aa)	Tag	Expression System
pFL-pUCDM:FL ARISC WT	Human	codon-optimised (GenScript)	BRCC36-Abraxas1	1-316 (BRCC36); 1-409 (Abraxas1)	N-dStrepII (Abraxas1)	BRCC45-MERIT40	1-383 (BRCC45); 1-329 (MERIT40)	N-6xHis (BRCC45)	*Sf9*
pFL-pUCDM:FL BRISC WT	Human	codon-optimised (GenScript)	BRCC36-Abraxas2	1-316 (BRCC36); 1-415 (Abraxas2)	N-dStrepII (Abraxas2)	BRCC45-MERIT40	1-383 (BRCC45); 1-329 (MERIT40)	N-6xHis (BRCC45)	*Sf9*
pFL-pUCDM:ARISCΔN WT	Human	cloning (see **Supplementary Table 3**)	BRCC36-Abraxas1	1-316 (BRCC36); 1-409 (Abraxas1)	none	BRCC45-MERIT40ΔN	1-383 (BRCC45); 72-329 (MERIT40ΔN)	N-6xHis (BRCC45)	*Sf9*
pFL-pUCDM:ARISCΔC WT	Human	cloning (see **Supplementary Table 3**)	BRCC36-Abraxas1ΔC	1-316 (BRCC36); 1-265 (Abraxas1ΔC)	none	BRCC45-MERIT40	1-383 (BRCC45); 1-329 (MERIT40)	N-6xHis (BRCC45)	*Sf9*
pFL-pUCDM:ARISCΔNΔC WT	Human	cloning (see **Supplementary Table 3**)	BRCC36-Abraxas1ΔC	1-316 (BRCC36); 1-265 (Abraxas1ΔC)	none	BRCC45-MERIT40ΔN	1-383 (BRCC45); 72-329 (MERIT40ΔN)	N-6xHis (BRCC45)	*Sf9*
pFL-pUCDM:FL ARISC E33A	Human	codon-optimised (GenScript)	BRCC36 E33A-Abraxas1	1-316 (BRCC36 E33A); 1-409 (Abraxas1)	N-dStrepII (Abraxas1)	BRCC45-MERIT40	1-383 (BRCC45); 1-329 (MERIT40)	N-6xHis (BRCC45)	*Sf9*
pFL-pUCDM:FL ARISC E33A$^{\text{BRCC36(S98K)}}$	Human	codon-optimised (GenScript)	BRCC36 E33A+S98K-Abraxas1	1-316 (BRCC36 E33A+S98K); 1-409 (Abraxas1)	N-dStrepII (Abraxas1)	BRCC45-MERIT40	1-383 (BRCC45); 1-329 (MERIT40)	N-6xHis (BRCC45)	*Sf9*
pFL-pUCDM:ARISC E33A$^{\text{Abraxas1(Δ42-55)}}$	Human	codon-optimised (GenScript)	BRCC36 E33A-Abraxas1 Δ42-55	1-316 (BRCC36 E33A); 1-409 (Abraxas1 Δ42-55)	N-dStrepII (Abraxas1)	BRCC45-MERIT40	1-383 (BRCC45); 1-329 (MERIT40)	N-6xHis (BRCC45)	*Sf9*
pFL-pUCDM:ARISC E33A$^{\text{BRCC45(ΔLoop)}}$	Human	codon-optimised (GenScript)	BRCC36 E33A-Abraxas1	1-316 (BRCC36 E33A); 1-409 (Abraxas1)	N-dStrepII (Abraxas1)	BRCC45 ΔLoop-MERIT40	1-383/Δ161-170 (BRCC45 ΔLoop); 1-329 (MERIT40)	N-6xHis (BRCC45)	*Sf9*
pFL-pUCDM:ARISCΔC E33A	Human	codon-optimised (GenScript)	BRCC36 E33A-Abraxas1ΔC	1-316 (BRCC36 E33A); 1-265 (Abraxas1ΔC)	N-dStrepII (Abraxas1)	BRCC45-MERIT40	1-383 (BRCC45); 1-329 (MERIT40)	N-6xHis (BRCC45)	*Sf9*
pFL-pUCDM:FL ARISC S98K	Human	codon-optimised (GenScript)	BRCC36 S98K-Abraxas1	1-316 (BRCC36 S98K); 1-409 (Abraxas1)	N-dStrepII (Abraxas1)	BRCC45-MERIT40	1-383 (BRCC45); 1-329 (MERIT40)	N-6xHis (BRCC45)	*Sf9*
pFL-pUCDM:FL ARISC S132A	Human	codon-optimised (GenScript)	BRCC36 S132A-Abraxas1	1-316 (BRCC36 S132A); 1-409 (Abraxas1)	N-dStrepII (Abraxas1)	BRCC45-MERIT40	1-383 (BRCC45); 1-329 (MERIT40)	N-6xHis (BRCC45)	*Sf9*
pFL-pUCDM:FL ARISC V134K	Human	codon-optimised (GenScript)	BRCC36 V134K-Abraxas1	1-316 (BRCC36 V134K); 1-409 (Abraxas1)	N-dStrepII (Abraxas1)	BRCC45-MERIT40	1-383 (BRCC45); 1-329 (MERIT40)	N-6xHis (BRCC45)	*Sf9*
pFL-pUCDM:FL ARISC M145A	Human	codon-optimised (GenScript)	BRCC36 M145A-Abraxas1	1-316 (BRCC36 M145A); 1-409 (Abraxas1)	N-dStrepII (Abraxas1)	BRCC45-MERIT40	1-383 (BRCC45); 1-329 (MERIT40)	N-6xHis (BRCC45)	*Sf9*
pFL-pUCDM:ARISC Δ42-55	Human	codon-optimised (GenScript)	BRCC36-Abraxas1 Δ42-55	1-316 (BRCC36); 1-409/Δ42-55 (Abraxas1 Δ42-55)	N-dStrepII (Abraxas1)	BRCC45-MERIT40	1-383 (BRCC45); 1-329 (MERIT40)	N-6xHis (BRCC45)	*Sf9*
pFL-pUCDM:FL ARISC F140A	Human	codon-optimised (GenScript)	BRCC36-Abraxas1	1-316 (BRCC36); 1-409 (Abraxas1)	N-dStrepII (Abraxas1)	BRCC45 F140A-MERIT40	1-383 (BRCC45 F140A); 1-329 (MERIT40)	N-6xHis (BRCC45)	*Sf9*
pFL-pUCDM:ARISC ΔLoop	Human	codon-optimised (GenScript)	BRCC36-Abraxas1	1-316 (BRCC36); 1-409 (Abraxas1)	N-dStrepII (Abraxas1)	BRCC45 ΔLoop-MERIT40	1-383/Δ161-170 (BRCC45 ΔLoop); 1-329 (MERIT40)	N-6xHis (BRCC45)	*Sf9*
pFL-pUCDM:FL ARISC W165A	Human	codon-optimised (GenScript)	BRCC36-Abraxas1	1-316 (BRCC36); 1-409 (Abraxas1)	N-dStrepII (Abraxas1)	BRCC45 W165A-MERIT40	1-383 (BRCC45 W165A); 1-329 (MERIT40)	N-6xHis (BRCC45)	*Sf9*
pFL-pUCDM:FL ARISC Y263A+R320A	Human	codon-optimised (GenScript)	BRCC36-Abraxas1	1-316 (BRCC36); 1-409 (Abraxas1)	N-dStrepII (Abraxas1)	BRCC45 Y263A+R320A-MERIT40	1-383 (BRCC45 Y263A+R320A); 1-329 (MERIT40)	N-6xHis (BRCC45)	*Sf9*

RAP80 variants

Expression Construct	Species	Cloning/Gene Synthesis	Protein/Sub-complex 1 (name)	Protein/Sub-complex 1 (aa)	Tag	Protein/Sub-complex 2 (name)	Protein/Sub-complex 2 (aa)	Tag	Expression System
pFastBac-HTB:FL RAP80 WT	Human	cloning (see **Supplementary Table 3**)	RAP80	1-719	N-6xHis	none	none	none	*Sf9*
pFastBac-HTB:RAP80 AIR	Human	cloning (see **Supplementary Table 3**)	RAP80 AIR	273-402	N-6xHis	none	none	none	*Sf9*
pFastBac-HTB:RAP80 ΔUIMs	Human	cloning (see **Supplementary Table 3**)	RAP80 ΔUIMs	1-719/Δ74-131	N-6xHis	none	none	none	*Sf9*
pFastBac-HTB:RAP80 ΔZnF	Human	cloning (see **Supplementary Table 3**)	RAP80 ΔZnF	1-719/Δ499-588	N-6xHis	none	none	none	*Sf9*

Ubiquitin variants

Expression Construct	Species	Cloning/Gene Synthesis	Protein/Sub-complex 1 (name)	Protein/Sub-complex 1 (aa)	Tag	Protein/Sub-complex 2 (name)	Protein/Sub-complex 2 (aa)	Tag	Expression System
pProEx-HTB:Cys0-ubiquitin K63R	Human	cloning (see **Supplementary Table 3**)	Cys0-ubiquitin K63R	1-76	N-6xHis	none	none	none	*E. coli*
pET3a:ubiquitin R74C ΔGG	Human	codon-optimised (GenScript)	ubiquitin R74C ΔGG	1-74	none	none	none	none	*E. coli*
pET3a:ubiquitin K63R	Human	codon-optimised (GenScript)	ubiquitin K63R	1-76	N-8xHis	none	none	none	*E. coli*

Supplementary Table 3: List of primers

Name	Sequence
FL BRCC36 WT_pFL_F	TATCCATGGGCCATGGCGGTGCAGGTG
FL BRCC36 WT_pFL_R	TTAATGCATTTATTCTAGAGAAGAAAGTTCTTG
FL Abraxas1 WT_pFL_F	CGCGTCGACACCATGGAGGGGGAGAGT
FL Abraxas1 WT_pFL_R	TCAAAGCTT TCAAAATGTAGGAGACCGTGA
Abraxas1ΔC_pFL_F	ATTCAGGCAGCATGATGAAGAGAGAAGAACATCCAAAAAGACCCTCAG
Abraxas1ΔC_pFL_R	GTTCTTCTCTCTTCATCATGCTGCCTGAATCTGTGCTCCTCTCCTTTT
FL BRCC45 WT_pUCDM_F	TATGGATCCACCATGGCCCATCACCATCACCATCACGAT
FL BRCC45 WT_pUCDM_R	CTAGTCGACCTAGAGCTTTCCATTGGCAAATGCTGCCTC
FL MERIT40 WT_pUCDM_F	TATCCCGGGACCATGGAAGTGGCAGAG
FL MERIT40 WT_pUCDM_R	TCAATGCATTCAGACAGTGGCCTCAACCTCAAT
MERIT40ΔN_pUCDM_F	TATCCCGGGACCATGTCCTGGCAGGTG
MERIT40ΔN_pUCDM_R	TCAATGCATTCAGACAGTGGCCTCAACCTCAAT
FL RAP80 WT_pFastBac-HTB_F	CGCGGATCCATGCCACGGAGAAAG
FL RAP80 WT_pFastBac-HTB_R	TCAGTCGACTCAGAATTTTCTCCTTCT
RAP80 AIR_pFastBac-HTB_F	GGATCC GGCGCC atgGGCACTGTGAACTATTTCTGGGGTA
RAP80 AIR_pFastBac-HTB_R	tataGTCGACtCaTTaGGAAGACTGACCATGACTGGTTGTTG
RAP80 ΔUIMs_pFastBac-HTB_F	AAGTGTTTGGCCAAACGACCTCTGGCCACTGGACCGTCTTCCCAG
RAP80 ΔUIMs_pFastBac-HTB_R	AGTGGCCAGAGGTCGTTTGGCCAAACACTTTGCTCTATTCGACTG
RAP80 ΔZnF_pFastBac-HTB_F	ATTTCTACCTTCTCAGACCAAGGAGATGGACCTGAAGGGAGTGGA
RAP80 ΔZnF_pFastBac-HTB_R	TCCATCTCCTTGGTCTGAGAAGGTAGAAATAGCTACTTCCTTCTC
Cys0-ubiquitin WT_F	GCCATGGGATGCATGCAGATCTTCGTCAAGACGTTAACCGGT
Cys0-ubiquitin WT_R	GATCTGCATGCATCCCATGGCGCCCTGAAAATACAGGTTTTC
Cys0-ubiquitin K63R_F	AACATTCAGAGAGAGTCGACCTTACATCTTGTCTTAAGACTA
Cys0-ubiquitin K63R_R	GGTCGACTCTCTCTGAATGTTGTAATCAGACAGCGTTCTACC

Supplementary References

1. Berezein, C. *et al*. ConSeq: the identification of functionally and structurally important residues in protein sequences. *Bioinformatics* **20**, 1322–4 (2004).
