# Supplementary material for: Mechanism of K63-linked polyubiquitin recognition and cleavage by the BRCA1-A complex
Source: Nat Commun. 2026 Aug 11;17:8091. doi: 10.1038/s41467-026-75795-y (PMC13462971; doi:10.1038/s41467-026-75795-y)
Supplement: Supplementary file 2 [file 41467_2026_75795_MOESM2_ESM.pdf]

nature portfolio

Corresponding author(s): Elton Zeqiraj

Last updated by author(s): 06.07.2026

Reporting Summary

Nature Portfolio wishes to improve the reproducibility of the work that we publish. This form provides structure for consistency and transparency in reporting. For further information on Nature Portfolio policies, see our Editorial Policies and the Editorial Policy Checklist.

Statistics

For all statistical analyses, confirm that the following items are present in the figure legend, table legend, main text, or Methods section.

n/a	Confirmed	
☐	☒	The exact sample size (n) for each experimental group/condition, given as a discrete number and unit of measurement
☐	☒	A statement on whether measurements were taken from distinct samples or whether the same sample was measured repeatedly
☐	☒	The statistical test(s) used AND whether they are one- or two-sided<br>*Only common tests should be described solely by name; describe more complex techniques in the Methods section.*
☐	☒	A description of all covariates tested
☒	☐	A description of any assumptions or corrections, such as tests of normality and adjustment for multiple comparisons
☐	☒	A full description of the statistical parameters including central tendency (e.g. means) or other basic estimates (e.g. regression coefficient) AND variation (e.g. standard deviation) or associated estimates of uncertainty (e.g. confidence intervals)
☐	☒	For null hypothesis testing, the test statistic (e.g. F, t, r) with confidence intervals, effect sizes, degrees of freedom and P value noted<br>*Give P values as exact values whenever suitable.*
☒	☐	For Bayesian analysis, information on the choice of priors and Markov chain Monte Carlo settings
☒	☐	For hierarchical and complex designs, identification of the appropriate level for tests and full reporting of outcomes
☒	☐	Estimates of effect sizes (e.g. Cohen's d, Pearson's r), indicating how they were calculated

Our web collection on statistics for biologists contains articles on many of the points above.

Software and code

Policy information about availability of computer code

Data collection	Cryo-EM data were collected using ThermoFisher EPU software (v3.8). Measurements of spectral shift assays were performed using DI.Control software (v2.1.1; NanoTemper Technologies).
Data analysis	Cryo-EM data and models were analyzed using RELION (v4.0), cryoSPARC (v4.5.3), crYOLO (v1.6.1), Coot (v0.9.8.1), and PHENIX (v1.17.1). DUB activity assays (IQF) were analyzed using Prism 10 (v10.1.0); quantifications of gel-based ubiquitin chain cleavage assays were plotted using Prism 10 (v10.1.0). Data from spectral shift assays were analysed using DI.Screening Analysis software (v2.1.1; NanoTemper Technologies), and binding affinity curves were plotted in Prism 10 (v10.1.0). Intact mass spectrometry data were processed using UniDec. Cross-linking mass spectrometry data were collected and analysed using Xcalibur (v4.7), Tune (v4.2), and Proteome Discoverer (v3.0), and cross-linking results were visualised using xiView. IRIF were quantified and analyzed using CellProfiler (v4.2.5) and Prism 10 (v10). Structural representations were created in PyMOL (v3.1.3.1) or UCSF Chimera X (v1.6); additional structural analyses were performed using the AlphaFold 3 webserver (https://alphafoldserver.com/), the program Superpose, or the European Bioinformatics Institute (EBI) web service of the PISA (Proteins, Interfaces, Structures and Assemblies) software (https://www.ebi.ac.uk/pdbe/pisa/). Sequence alignment was performed with Clustal Omega (https://www.ebi.ac.uk/jdispatcher/msa/clustalo), and edited and displayed using the Consurf web server (https://consurf.tau.ac.il/consurf_index.php). Schematics depicted in Fig. 1a,c-g, Fig. 2d-h, Fig. 4a,f, Fig. 5f-i, Fig. 6, Supplementary Fig. 1b-e,g, Supplementary Fig. 2a,b, Supplementary Fig. 3, Supplementary Fig. 4b-d, Supplementary Fig. 5b,f, Supplementary Fig. 9b, Supplementary Fig. 10b, and Supplementary Fig. 11b,e were created in UCSF Chimera X (v1.6), the final model depicted in Fig. 8 was created using BioRender (https://www.biorender.com/), and all figure panels were assembled using Affinity Designer (v2).

For manuscripts utilizing custom algorithms or software that are central to the research but not yet described in published literature, software must be made available to editors and reviewers. We strongly encourage code deposition in a community repository (e.g. GitHub). See the Nature Portfolio guidelines for submitting code & software for further information.

Data

Policy information about availability of data

All manuscripts must include a data availability statement. This statement should provide the following information, where applicable:

- Accession codes, unique identifiers, or web links for publicly available datasets
- A description of any restrictions on data availability
- For clinical datasets or third party data, please ensure that the statement adheres to our policy

Cryo-EM maps and associated structural models have been deposited to the Electron Microscopy Data Bank and Protein Data Bank under accession codes EMD-55122, EMD-55090, EMD-55069, EMD-55070, EMD-55071, EMD-55072, EMD-55073, EMD-55074, and PDBid: 9SQY (for the ARISC(E33A)–RAP80:K63-Ub7 complex); EMD-55119, EMD-55079, EMD-55080, EMD-55081, EMD-55077, EMD-55082, and PDBid: 9SQW (for the ARISCΔC(E33A):K63-Ub7 complex); EMD-55118, EMD-55085, EMD-55086, EMD-55088, EMD-55089, and PDBid: 9SQV (for the ARISCΔC(E33A):K63-Ub4 complex). Raw cross-linking mass spectrometry data have been deposited to the ProteomeXchange Consortium via the PRIDE partner repository with the dataset identifier PXD068769. Reviewers can access the deposited dataset by using the following login details: username = reviewer_pxd068769@ebi.ac.uk; password = EYr4Tv9fayH8. All unique reagents are available upon request. Source data are provided with this paper.

Research involving human participants, their data, or biological material

Policy information about studies with human participants or human data. See also policy information about sex, gender (identity/presentation), and sexual orientation and race, ethnicity and racism.

Reporting on sex and gender	N/A
Reporting on race, ethnicity, or other socially relevant groupings	N/A
Population characteristics	N/A
Recruitment	N/A
Ethics oversight	N/A

Note that full information on the approval of the study protocol must also be provided in the manuscript.

Field-specific reporting

Please select the one below that is the best fit for your research. If you are not sure, read the appropriate sections before making your selection.

☒ Life sciences ☐ Behavioural & social sciences ☐ Ecological, evolutionary & environmental sciences

For a reference copy of the document with all sections, see nature.com/documents/nr-reporting-summary-flat.pdf

Life sciences study design

All studies must disclose on these points even when the disclosure is negative.

Sample size	Cryo-EM data were collected for a total of approximately 2-4 days. Sample size for cryo-EM analysis was arbitrary chosen depending on sample and instrument availability, and to ensure a sufficient number of particles to obtain structures at the reported resolutions.
Data exclusions	No data exclusion.
Replication	All data was replicated at least two times successfully and in line with accepted scientific standards.
Randomization	No randomization during allocation into experimental groups was performed. Sample selection bias and/or accidental bias was addressed by use of appropriate controls.
Blinding	Structure determination was performed without blinding. The sample identity was known to the investigators as this was required for appropriate interpretation of EM maps and models.

Reporting for specific materials, systems and methods

We require information from authors about some types of materials, experimental systems and methods used in many studies. Here, indicate whether each material, system or method listed is relevant to your study. If you are not sure if a list item applies to your research, read the appropriate section before selecting a response.

Materials & experimental systems

n/a	Involved in the study	
☐	☒	Antibodies
☐	☒	Eukaryotic cell lines
☒	☐	Palaeontology and archaeology
☒	☐	Animals and other organisms
☒	☐	Clinical data
☒	☐	Dual use research of concern
☒	☐	Plants

Methods

n/a	Involved in the study	
☒	☐	ChIP-seq
☒	☐	Flow cytometry
☒	☐	MRI-based neuroimaging

Antibodies

Antibodies used

1. Abraxas1 (Bethyl laboratories; catalogue number: A302-180A); dilution 1:1000 for Western Blotting
2. BRCC36 (Abcam; catalogue number: ab108411); dilution 1:1000 for Western Blotting
3. GAPDH (Cell Signaling; catalogue number: 2118S); dilution 1:5000 for Western Blotting
4. anti-HA.11 Epitope Tag (BioLegend; catalogue number: 901516); dilution 1:500 for immunofluorescence
5. Phospho-Histone H2A.X (Ser139) (Cell Signaling; catalogue number: 9718S); dilution 1:500 for immunofluorescence
6. anti-mouse AlexaFluor 488-conjugated secondary antibody (Thermo Scientific; catalogue number: A-11029); dilution 1:500 for immunofluorescence
7. anti-rabbit AlexaFluor 647-conjugated secondary antibody (Thermo Scientific; catalogue number: A-21245); dilution 1:500 for immunofluorescence
8. anti-rabbit HRP-conjugated (Amersham; catalogue number: NA934); dilution 1:2000 for Western Blotting
9. BRCC45 (Abcam; catalogue number: ab177960); dilution 1:1000 for Western Blotting
10. anti-FLAG antibody (Cell Signaling; catalogue number: 14793); dilution 1:400 for immunofluorescence

Validation

Validation of all the commercially purchased antibodies can be found on their respective websites outlined below.

1. https://www.fortislife.com/products/primary-antibodies/rabbit-anti-abraxas-antibody/BETHYL-A302-180?selected=A302-180A
2. https://www.abcam.com/en-us/products/primary-antibodies/brcc36-antibody-epr4366-ab108411?srsltid=AfmBOoqupX6FAbTbnMNwPaqZxY9dwf2zgd8-sdoudQVpeTbsu0qQQZIO
3. https://www.cellsignal.com/products/2118/datasheet?images=1&srsltid=AfmBOooG0ZLV8Xjd21bQrmW5_LPjcnTABGyT35dzbrnRU32kHBSf__ow
4. https://www.biolegend.com/en-gb/products/anti-ha-11-epitope-tag-antibody-11071
5. https://www.cellsignal.com/products/primary-antibodies/phospho-histone-h2a-x-ser139-20e3-rabbit-mab/9718?srsltid=AfmBOoodyf6cFVYnWd2TmSKuAi9EEVzTMxFy9x-RsBLKLlayRr4DEQze
6. https://www.thermofisher.com/antibody/product/Goat-anti-Mouse-IgG-H-L-Highly-Cross-Adsorbed-Secondary-Antibody-Polyclonal/A-11029
7. https://www.thermofisher.com/antibody/product/Goat-anti-Rabbit-IgG-H-L-Highly-Cross-Adsorbed-Secondary-Antibody-Polyclonal/A-21245
8. https://www.cytivalifesciences.com/en/us/products/items/amersham-ecl-hrp-conjugated-antibodies-p-06260?selectedProduct=25800685\
9. https://www.abcam.com/en-us/products/primary-antibodies/brcc45-bre-antibody-epr11858-ab177960
10. https://www.cellsignal.com/products/primary-antibodies/dykddddk-tag-d6w5b-rabbit-monoclonal-antibody-binds-to-same-epitope-as-sigma-aldrich-anti-flag-m2-antibody/14793

Eukaryotic cell lines

Policy information about cell lines and Sex and Gender in Research

Cell line source(s)	Sf9 cells used for viruses generation and protein expression were obtained from Thermo Fisher Scientific. HT-29 and HEK293T cell lines were purchased from ATCC.
Authentication	No authentication of Sf9 cell line was performed in our laboratory. HT-29 and HEK293T cell lines authentication was performed by ATCC.
Mycoplasma contamination	Regular test are held in our laboratory (every 2 months). No mycoplasma contamination was detected.
Commonly misidentified lines (See ICLAC register)	Commonly misidentified lines were not used.

Plants

Seed stocks	N/A
Novel plant genotypes	N/A
Authentication	N/A
