# Supplementary material for: Mechanism of K63-linked polyubiquitin recognition and cleavage by the BRCA1-A complex
Source: Nat Commun. 2026 Aug 11;17:8091. doi: 10.1038/s41467-026-75795-y (PMC13462971; doi:10.1038/s41467-026-75795-y)
Supplement: Supplementary file 3 [file 41467_2026_75795_MOESM3_ESM.pdf]

nature portfolio

Peer Review File

Mechanism of K63-linked polyubiquitin recognition and cleavage by the BRCA1-A complex

Corresponding Author: Professor Elton Zeqiraj

This file contains all reviewer reports in order by version, followed by all author rebuttals in order by version.

Version 0:

Reviewer comments:

Reviewer #1

(Remarks to the Author)
ARISC is a nuclear protein complex formed by a dimer of heterotetramers of the JAMM/MPN-type deubiquitinating enzyme (DUB) BRCC36, Abraxas1, BRCC45 and MERIT40; it has deubiquitinating activity against K63-linked polyUb. RAP80 is an accessory subunit of ARISC. ARISC-RAP80 has been shown to accumulate at ionizing radiation-induced foci (IRIF) through RAP80 interactions with SUMO and K63-linked polyUb chains at damage chromatin where it mediates the recruitment of the E3 ligase heterodimer BRCA1/BARD1 to DNA breaks via phospho-Abraxas1 binding to BRCA1 (BRCA1-A complex).
Here, Foglizzo et al. used biochemical and structural approaches to characterize the activity of the ARISC-RAP80 complex with K63-linked polyUb (n = 2-9) and used cell-based experiments to evaluate the contributions of individual ARISC-RAP80 functional domains to BRCA1-A accumulation at ionizing radiation-induced foci (IRIF). Activity assays using recombinant ARISC and ARISC-RAP80 complexes with K63-polyUb (Ub2-Ub9) showed that both ARISC and ARISC-RAP80 prefer longer chains (≥Ub4) and that ARISC-RAP80 appears to more efficiently cleave K63-chains of Ub6 and longer. Curiously, cyclic versions of the chains appear to be somewhat better substrates than more conventional linear K63-polyUb. In agreement with Rabl et al. (2019), ARISC and ARISC-RAP80 seem to preferentially cleave K63-polyUb chains from the distal end, although the authors provide convincing evidence for cleavage within the middle of chains (i.e., endo-DUB activity) as well.
A major thrust of the paper is to understand ARISC and ARISC-RAP80 interactions with K63-polyUb. For this purpose, cryo-EM and chemical (DSBU) cross-linking coupled with MS were employed. The cryo-EM structure of the ARISC-RAP80:K63-Ub7 complex suggests that K63-polyUb bound by ARISC-RAP80 adopts a compact conformation that has not been seen previously for K63-polyUb. Whereas bound P1' (proximal Ub) and P1 (distal Ub) adopt an extended conformation at the BRCC36 catalytic site, Ub (P1), Ub (P2), Ub (P3) and Ub (P4) interact with accessory Ub binding domains in BRCC36, Abraxas1 and BRCC45; these were proposed to promote a closed conformation to the K63-polyUb substrate. The N- and C-terminal regions of RAP80 were not visualized in the structure; this is disappointing as the preferential cleavage of longer K63-polyUb might have been explained by interactions of RAP80's N-terminal UIMs with substrate Ub moieties. Nonetheless, the cross-linking showed interactions between RAP80 N-terminus and Ub in K63-polyUb (n≥3) and between RAP80 C-terminus and Ub in K63-Ub5 and K63-Ub7.
DUB assays with ARISC or ARISC-RAP80 reconstituted with BRCC36, Abraxas1 and BRCC45 mutants selected based on the cryo-EM structures and cross-linking results were done to test the impact of the proposed individual UBD:Ub interactions on ARISC/ARISC-RAP80 activity. Cell-based assays with BRCC36 and Abraxas1 KO cells complemented with BRCC36 and Abraxas1 mutants revealed the contribution of these BRCC36:Ub and Abraxas1:Ub interactions to BRCA1-A accumulation at IRIF.
Overall, the paper provides substantial new information about ARISC-RAP80, which plays an important but poorly understood role in the DNA damage response mediated by BRCA1. However, some of the results are puzzling and deserve clarification and, ideally, additional data. Specific questions and comments are below.
1. Comparisons of the various K63-polyUb chains as substrates for cleavage by different ARISC complexes are difficult to interpret. One problem is the presentation of the gel-based assays: the reader is expected to make conclusions based on "eyeballing" relative band intensities; instead, a quantitative readout of these assays should be presented. Another problem is that, from the information given, one doesn't know if apparent differences in activity are due to substrate binding or Km, or kcat. In the context of trying to decipher the roles of the several different Ub interaction surfaces presented by ARISC-RAP80 subunits, this is important. At a minimum, the authors should show that the assays were done with initial substrate concentrations well below saturation to better compare catalytic efficiencies. Ideally, additionally determining initial rates with saturating substrates would be done to allow the results to be interpreted in terms of substrate binding.

2. K63-polyUb binding determination by more direct approaches than activity assays (i.e., as in #1 above) are warranted. The authors' structural data make a strong case that there are several distinct Ub interaction sites in ARISC-RAP80, and they report effects of mutating or otherwise eliminating some of those sites on both in vitro DUB activity and recruitment to DNA damage foci. However, there is no quantitative assessment of the relative contributions of those sites. I consider this a major deficiency in an otherwise strong study. Such data would go far to dispel some uncertainties about how to interpret the results (e.g., see #3 below).
3. That inactive BRCC36 (E33A) showed significantly reduced (ARISC-RAP80) recruitment to IRIF damage foci (Fig. 3g; Extended Fig. 5c) was attributed (line 503) to reduced polyUb binding. Given that there are several other interactions that can engage K63-polyUb, that is quite surprising. To support the authors' contention, in vitro binding (point #2) would be very useful. Another possibility that should be explored is that the ARISC mutations that reduced ARISC-RAP80 at IRIF had their effects by lowering levels of assembled ARISC-RAP80 rather than through reduced polyUb binding. Experiments to evaluate levels of the ARISC-RAP80 complex would address this. Some characterization of RAP80, MERIT40 and BRCC45 levels in BRCC36 E33A knock-in cell lines were reported in Jiang et al. (JCB, 2022), but the effect on the assembled complex is unclear.
4. The results from comparison of linear and cyclic K63-polyUb are a bit confusing and not wholly convincing. First and foremost, gel-based evidence that cycK63-polyUb chains are cleaved more efficiently than the linK63-polyUb counterparts warrant quantitation and evidence of reproducibility. Second, the authors apparently did not consider that the activity against the cyclic chains might be even greater than it appears in the gels; whereas a single cut in a linear substrate would generate smaller (poly)Ub fragments, reducing the size of a cyclic chain would require two cuts. Third, the authors might consider that a reason cyclic chains could be preferred is that, unlike with linear polyUb, whichever Ub units occupy the P1 and P1' sites all the remaining Ub distal and proximal to the cleavage site will be in the same register. Fourth, the distal-end preference for cleavage by the ARISC DUB (p. 24) seems inconsistent with the claimed preference for cyclic chains (which necessarily undergo endo-cleavage). Finally, the authors speculate (line 892) about the physiological function of the cycK63-polyUb; they should at least acknowledge that such chains may be an artifact of their in vitro chain synthesis reaction and are inconsequential in cells.
5. ARISC DUB activity was reduced when N-terminal MERIT40 was deleted (ARISCΔN), but little effect was observed with deletion of C-terminal Abraxas1 (ARISCΔC) or N-terminal MERIT40 + C-terminal Abraxas (ARISCΔNΔC). Shouldn't (ARISCΔNΔC) affect catalytically activity similarly to ARISCΔN?
6. Regarding BRCC45 UEV-C interactions with Ub seen in the cryo-EM structure and predicted by AlphaFold: does mutating BRCC45 UEV-C have any effect on BRCA1-A accumulation at IRIF?
7. (line 367) Given the prominent role played by RAP80 in polyUb binding and the increased activity of ARISC-RAP80 over ARISC, activity assays should include ARISC-RAP80 with the UIMs mutated to evaluate effects on longer-chain substrates. Along these lines, does addition of full-length RAP80 to ARISC skew the position of the first cleavage with long-chain substrates?
8. In the Fig. 4 legend (lines 691-694), the title says the cryo-EM structures are for ARISCΔC(E33A):K63-Ub7 and ARISCΔC(E33A):K63-Ub4 complexes, but the legends for 4a and 4b say ARISC(E33A)-RAP80:K63-Ub7 and ARISC(E33A)-RAP80:K63-Ub7

Reviewer #2

(Remarks to the Author)
In this manuscript, Foglizzo et al present a powerful structural and biochemical analysis of substrate recognition by ARISC-RAP80 (also known as BRCA1-A) metallo deubiquitinase (Dub) complex that regulates the recruitment of the larger BRCA1 supercomplex to ubiquitinated foci at damaged DNA sites and DSB repair within chromatin. Using enzymatically produced K63-linked polyubiquitin chains as substrates they managed to capture the structure of substrate bound state of the ARISC-RAP80 complex using single-particle cryo-EM at relatively high resolution. The structure allowed visualization of a larger piece of the polyubiquitin chain substrate bound to the complex revealing K63-isopeptide linked diubiquitin in the active site of the BRCC36 Dub catalytic unit in a productive orientation, with additional ubiquitins occupying previously unexpected sites on the complex beyond the Brcc36 catalytic unit.
The structural results were supported by solution crosslinking mass spectrometry, which identifies additional ubiquitin interaction sites distal to the catalytic core where ubiquitin binding contributes to catalytic efficiency. Guided by structure the authors created and validated mutants using both in vitro deubiquitinase assays and cell-based assays assessing BRCA1-A recruitment to DSBs. Overall, the study represents a significant mechanistic advance in understanding how a multi-protein nuclear DUB complex recognizes and processes polyubiquitin chains in the DNA damage response pathway. The cryo-EM work is rigorous with solid the structural interpretation, and the manuscript is clearly written with high-quality figures in line with expectations for a paper in Nature Communications.
Specific Comments:
(1) The authors have prepared a stochiometric complex of ARISC with full-length RAP80 and mixed it with cyclic Ub7 polymer for cryo-EM reconstruction. The cyclic substrate seems better than the corresponding linear polyubiquitin substrate, so it makes good sense to use it for cryo-EM studies. The observed ubiquitin groups at positions in BRCC36 active site (P1'-P1) align well with the expected positions of the same sites in AMSH-LP, a prototypical JAMM family Dub. Two additional ubiquitin densities (P3 and P4) mapped onto ABRAXAS1 with high confidence, and the isopeptide linkages connecting these positions are well resolved.

The occupancy at these sites and the novel arrangement of ubiquitin groups in the K63-chain provides interesting

mechanistic insights. However, density corresponding to ubiquitin engagement at the BRCC45 UEV domains is absent, which could be perhaps due to lower affinity as these sites. It is plausible that the lack of ubiquitin density at the arms could be due to the constraints imposed by the non-native cyclic structure of the polyubiquitin chain. It would strengthen the conclusions if the authors included crosslinking mass spectrometry data collected using linear Ub7 chain (as an additional panel in Figure 2). With the long linear substrate like Ub7 there could be more crosslinks between ubiquitin and BRCC45 domains than what has been observed in Figure 2g. Please note that going from Ub4 (linear) to Ub7 (cyclic) there are hardly any more crosslinks from Ub to BRCC45 UEV domains (if not fewer) which does correlate with difference in catalytic activity between these substrates.

(2) The nature of cyclic structure has not been completely established. The loss of water (dehydration) is taken as evidence for cyclic structure, which is reasonable but not conclusive. They argue unanchored Lys63 linked chains have a propensity to form head-to-tail cyclic conformation bringing Lys63 of the first and G76 carboxylate of the last ubiquitin to proximity to be linked by the E2 enzyme. In this unexpected reaction, it is possible that the Met 1 alpha-amino group of the first Ub becomes linked to the last Gly76. Indeed, to prevent cyclization they introduce two modifications (blocking the N-terminus and mutating K63 to R). Since cryo-EM did not reveal the complete structure of the substrate, it is important to consider a different way of establishing the nature of the cyclic structure of the polyubiquitin chain substrate.

(3) The authors should consider validating P2 and P3 interfaces observed in their structure through the cell based IRIF assay (such as mutant of Abraxas1 E4).
(4) In the model presented in Figure 6g, would it not make more sense to connect the ubiquitin chain at the catalytic unit to that emanating from the nucleosome?

(5) The authors need to explain the mechanistic implication of their observation of only one of BRCC36 being occupied by ubiquitin. Does this asymmetry imply any functional relevance?

Reviewer #3

(Remarks to the Author)
I co-reviewed this manuscript with one of the reviewers who provided the listed reports. This is part of the Nature Communications initiative to facilitate training in peer review and to provide appropriate recognition for Early Career Researchers who co-review manuscripts.

Reviewer #4

(Remarks to the Author)
The manuscript from Foglizzo and colleagues entitled “Mechanism of K63-linked polyubiquitin recognition and cleavage by the BRCA1-A complex” described the structural and biochemical characterization of a deubiquitinase complex comprised of ARISC and RAP80 in complex with K63-linked polyubiquitn chains of different lengths. The ARISC complex functions in DNA damage signaling. This portion of this complex is itself comprised of protein subunits including Abraxis1, BRCC36, BRCC45 and MERIT40. The data show how ARISC-RAP80 engage K63 linked polyUb chains in a manner distinct from previous K63 interacting proteins, and perform appropriate follow up studies through mutagenesis of key residue to validate the conclusions biochemically and in cells. The authors take the data one step further and elucidate how these interactions between the DUB complex and K63 linked poly Ub are structurally and kinetically modulated by the length and configuration of the chain (linear vs circular). Finally, they conclude the paper by demonstrating the catalytic mechanism for the DUB activity, which acts on the distal Ub subunit within the K63 polyUB chain. Overall, the data in this paper are impressive and the interpretation of the results consistent with the observations. The work represents an important advance for the ubiquitin and DNA damage field. Below are a number of questions that arose during review of the manuscript that require clarification and/or suggestions for improving the impact of this work.

For the DUB catalytic assays in Fig 1c-e, the figure legend indicates that n=3 data were collected. I find the data very informative as is. That said, it would be additionally informative if certain aspects of the data were presented in a quantitative manner, whether it be for loss of the full length parental K63 chain, or formation of partially or completely disassembled Ub products of the assay.

Looking carefully at the data in Fig 1e, it appears that linear Ub5 chains are not fully processed (upper band eliminated), while the circular form appears to be more extensively processed. This surprised me given that the paper argues that circular Ub5-K63 chains were in fact refractory to cleavage as compared to linear chains or circular chains with additional subunits. What is the explanation for this?

Following on this point, it was not clearly described how the 6x-His and K63R mutated proteins, added to chain formation assays substoichiometrically, would yield different reaction products. Specifically, I could not discern how K63R mutant Ub could alter cyclization when added to a distal position. Taking this one step further, the authors provided circumstantial evidence of chain cyclization (through intact mass analysis), but I did not see any irrefutable direct evidence to show precisely the precise nature of the proposed cyclization. The authors should provide a more thorough description of what they do and do not know about the proposed cyclized chains being used in the assays, and if possible, generate data that directly demonstrates the position of cyclization. Is it possible that the cyclized chains represent a mixed population of

differentially linked products?
The data describe a novel Ub1-Ub3 interaction in the bent K63 polyUb chains engaged with ARISC-RAP80. Have the authors tested mutant forms of K63 polyUb that would not permit these interactions (e.g. text mentions positions the I44 hydrophobic patch and D58 acidic residue) to see whether these residues/interactions are required for K63-polyUb cleavage by ARISC-RAP80?

Have the authors tested whether linear polyUb chains linked through M1 will biochemically engage the ARISC-RAP80 complex. If these chains are modeled into the same position at the K63 chains tested here, what factors make them incompatible for engagement/proteolysis.

Is their a structural or biochemical reason to explain why no chemical crosslinks observed between BRCC36 and K63-poly Ub chains?

The authors should offer additional text to describe what the P1' ubiquitin is. DO they believe that a secondary K63 polyUb chain engages with a catalytically active ARISC-RAP80 complex as part of its normal function?

In Fig 3g, the authors test whether mutation of E33A and S98K of BRCC36 affect the formation of g-H2AX foci 4 hr post DNA damage through gamma irradiation. What additional mutations have been tested and do any show inhibition/enhancement of DUB activity? Text later in the paper speaks to BRCC45 residues including K270, K360 and K345, for example.The fact that these potentially engage with unbranched lysine residues on Ub itself if highly interesting.

As the authors were attempting to elucidate DUB catalytic mechanism versus the distal or proximal chain subunit, and given the differences between linear chains and the purported circular chains, I could not help but wonder if the paper would benefit from appending the K63-linked polyUb chain onto a substrate like peptide or protein to show the way that the ARISC-RAP80 complex orients a non-polyUb feature in relation to the chain itself. While this may be more work than is reasonable for this paper, it would be highly informative for the field.

Version 1:

Reviewer comments:

Reviewer #1

(Remarks to the Author)
In this revised manuscript, Greenberg, Zeqiraj, and their coauthors have comprehensively and satisfactorily addressed all concerns with the original submission. The authors are to be congratulated on their interesting and high-quality study.

Reviewer #2

(Remarks to the Author)
In this revised version of their manuscript, the authors have adequately addressed most of my concerns and comments which I believe has substantially improved the manuscript. The authors have performed additional experiments to the best of their abilities in response to the concerns raised by both this reviewer and the other reviewers, and these efforts have significantly strengthened the study to a level appropriate for publications in this journal. Restating my remarks from the earlier round of review, I believe the results presented in this report reveal unique insights into polyubiquitin recognition and chain processing, significantly advancing our understanding of structural basis of deubiquitination at DNA damage foci by the BRCA1-A associated deubiquitinase complex. I have no hesitation in recommending this manuscript for publication.

Reviewer #3

(Remarks to the Author)
I co-reviewed this manuscript with one of the reviewers who provided the listed reports. This is part of the Nature Communications initiative to facilitate training in peer review and to provide appropriate recognition for Early Career Researchers who co-review manuscripts.

Reviewer #4

(Remarks to the Author)
The authors have been responsive and have added meaningful new experiments, particularly the fluorescent substrate assays and linkage-selectivity DUB experiments. They have provided thoughtful responses to the queries from the various reviewers. I feel that the conclusions of the paper are well supported and that the limitations are appropriately managed appropriately..

Open Access This Peer Review File is licensed under a Creative Commons Attribution 4.0 International License, which permits use, sharing, adaptation, distribution and reproduction in any medium or format, as long as you give appropriate credit to the original author(s) and the source, provide a link to the Creative Commons license, and indicate if changes were made.
In cases where reviewers are anonymous, credit should be given to 'Anonymous Referee' and the source.
The images or other third party material in this Peer Review File are included in the article's Creative Commons license, unless indicated otherwise in a credit line to the material. If material is not included in the article's Creative Commons license and your intended use is not permitted by statutory regulation or exceeds the permitted use, you will need to obtain permission directly from the copyright holder.
To view a copy of this license, visit https://creativecommons.org/licenses/by/4.0/

Response to reviewer comments

We thank the reviewers for their constructure evaluation of our manuscript and provide a point-by-point response below.

Reviewer #1 (Remarks to the Author):

ARISC is a nuclear protein complex formed by a dimer of heterotetramers of the JAMM/MPN-type deubiquitinating enzyme (DUB) BRCC36, Abraxas1, BRCC45 and MERIT40; it has deubiquitinating activity against K63-linked polyUb. RAP80 is an accessory subunit of ARISC. ARISC-RAP80 has been shown to accumulate at ionizing radiation-induced foci (IRIF) through RAP80 interactions with SUMO and K63-linked polyUb chains at damage chromatin where it mediates the recruitment of the E3 ligase heterodimer BRCA1/BARD1 to DNA breaks via phospho-Abraxas1 binding to BRCA1 (BRCA1-A complex).

Here, Foglizzo et al. used biochemical and structural approaches to characterize the activity of the ARISC-RAP80 complex with K63-linked polyUb (n = 2-9) and used cell-based experiments to evaluate the contributions of individual ARISC-RAP80 functional domains to BRCA1-A accumulation at ionizing radiation-induced foci (IRIF). Activity assays using recombinant ARISC and ARISC-RAP80 complexes with K63-polyUb (Ub2-Ub9) showed that both ARISC and ARISC-RAP80 prefer longer chains (≥Ub4) and that ARISC-RAP80 appears to more efficiently cleave K63-chains of Ub6 and longer. Curiously, cyclic versions of the chains appear to be somewhat better substrates than more conventional linear K63-polyUb. In agreement with Rabl et al. (2019), ARISC and ARISC-RAP80 seem to preferentially cleave K63-polyUb chains from the distal end, although the authors provide convincing evidence for cleavage within the middle of chains (i.e., endo-DUB activity) as well.

A major thrust of the paper is to understand ARISC and ARISC-RAP80 interactions with K63-polyUb. For this purpose, cryo-EM and chemical (DSBU) cross-linking coupled with MS were employed. The cryo-EM structure of the ARISC-RAP80:K63-Ub7 complex suggests that K63-polyUb bound by ARISC-RAP80 adopts a compact conformation that has not been seen previously for K63-polyUb. Whereas bound P1' (proximal Ub) and P1 (distal Ub) adopt an extended conformation at the BRCC36 catalytic site, Ub (P1), Ub (P2), Ub (P3) and Ub (P4) interact with accessory Ub binding domains in BRCC36, Abraxas1 and BRCC45; these were proposed to promote a closed conformation to the K63-polyUb substrate. The N- and C-terminal regions of RAP80 were not visualized in the structure; this is disappointing as the preferential cleavage of longer K63-polyUb might have been explained by interactions of RAP80's N-terminal UIMs with substrate Ub moieties. Nonetheless, the cross-linking showed interactions between RAP80 N-terminus and Ub in K63-polyUb (n≥3) and between RAP80 C-terminus and Ub in K63-Ub5 and K63-Ub7.

DUB assays with ARISC or ARISC-RAP80 reconstituted with BRCC36, Abraxas1 and BRCC45 mutants selected based on the cryo-EM structures and cross-linking results were done to test the impact of the proposed individual UBD:Ub interactions on ARISC/ARISC-RAP80 activity. Cell-based assays with BRCC36 and Abraxas1 KO cells complemented with BRCC36 and Abraxas1 mutants revealed the contribution of these BRCC36:Ub and Abraxas1:Ub interactions to BRCA1-A accumulation at IRIF.

Overall, the paper provides substantial new information about ARISC-RAP80, which plays an important but poorly understood role in the DNA damage response mediated by BRCA1.

However, some of the results are puzzling and deserve clarification and, ideally, additional data. Specific questions and comments are below.

1. Comparisons of the various K63-polyUb chains as substrates for cleavage by different ARISC complexes are difficult to interpret. One problem is the presentation of the gel-based assays: the reader is expected to make conclusions based on “eyeballing” relative band intensities; instead, a quantitative readout of these assays should be presented. Another problem is that, from the information given, one doesn’t know if apparent differences in activity are due to substrate binding or Km, or kcat. In the context of trying to decipher the roles of the several different Ub interaction surfaces presented by ARISC-RAP80 subunits, this is important. At a minimum, the authors should show that the assays were done with initial substrate concentrations well below saturation to better compare catalytic efficiencies. Ideally, additionally determining initial rates with saturating substrates would be done to allow the results to be interpreted in terms of substrate binding.

We thank the reviewer for highlighting this point. Silver staining is highly sensitive but saturates quickly and has a narrow dynamic range, making it unreliable for band quantification[1,2]. We have therefore performed new activity assays using fluorescently labelled substrates in the revised manuscript. These assays and the related quantifications are included in **Fig. 1f**, **Fig. 6a,b**, and **Supplementary Fig. 1d,e**.

To fully address the reviewer’s comment, we assayed ARISC and ARISC–RAP80 against increased concentrations of our AF488-^{Cys}Ub4^{K63R} substrate (**Figure R1a,b**). Quantification of these experiments showed that both DUB complexes reach saturation at 1 µM of K63-Ub4 (**Figure R1c**), indicating that the activity assays reported in our initial manuscript, done at 5 nM or 10 nM of ARISC and ARISC–RAP80 complexes, were performed under saturating substrate conditions.

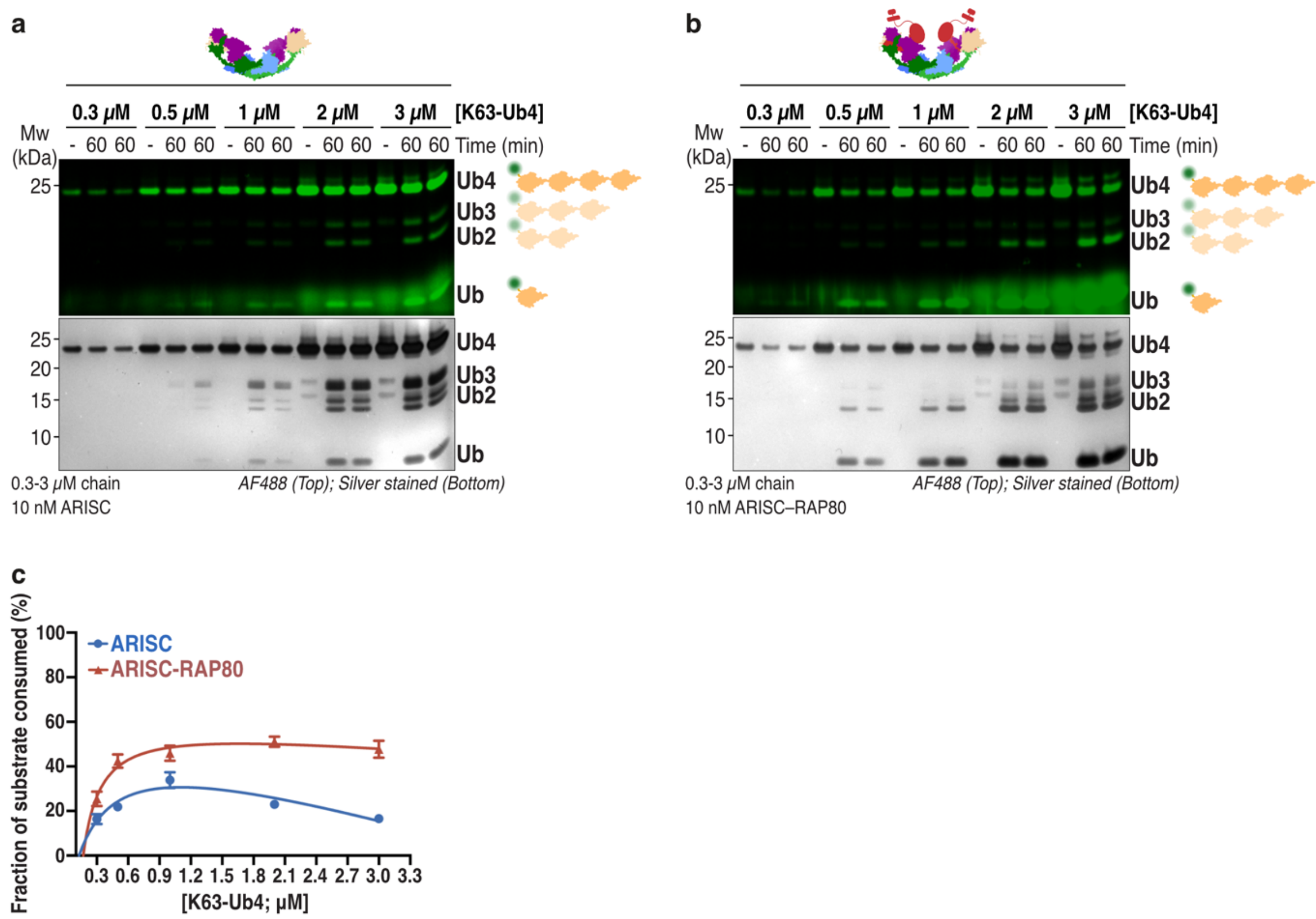

Figure R1. Determination of non-saturating and saturating substrate concentrations.
a,b, Gel-based ubiquitin chain cleavage assays. Alexa-Fluor (AF488) labelled distally (AF488-^{Cys}Ub4^{K63R}) blocked K63-Ub4 chains (0.3 µM-3 µM) were incubated with ARISC (**a**) or ARISC–RAP80 (**b**) (both at 10 nM) for 60 minutes. Cleavage activity was analysed by SDS-PAGE; gels were scanned using an iBright FL 1500 gel imaging system (excitation: 455-485 nm, emission: 508-557 nm) prior to being silver stained. Enzymatic reactions corresponding to two independent experiments were loaded side-by-side on the same gels. **c,** Quantifications of the enzymatic assays shown in **a,b**. The disappearance of the K63-Ub4 parent band was

quantified using densitometry, and plotted as fraction of substrate consumed (%). Data points are mean ± SEM of two independent experiments.

We subsequently compared the catalytic efficiencies and substrate binding abilities of ARISC and ARISC–RAP80 complexes (at 10 nM) against non-saturating (0.3 μM) and saturating (1.5 μM) concentrations of AF488-^{Cys}Ub4^{K63R} substrate, by quantifying the disappearance of the Ub4 parent band using densitometry (**Supplementary Fig. 1d,e**). We observed increased activity of ARISC–RAP80 compared to ARISC and ARISC–RAP80 AIR in the presence of non-saturating and saturating substrate. Likewise, ARISC and ARISC–RAP80 AIR displayed comparable enzymatic activities at both concentrations of K63-Ub4 (**Supplementary Fig. 1d,e**). Together, these data indicate that: 1) ARISC–RAP80 possess higher catalytic efficiency and ability to bind ubiquitin chains than ARISC and ARISC–RAP80 AIR; and that 2) in isolation, RAP80 AIR does not enhance ARISC activity nor contribute to ARISC-mediated ubiquitin chain binding.

2. K63-polyUb binding determination by more direct approaches than activity assays (i.e., as in #1 above) are warranted. The authors' structural data make a strong case that there are several distinct Ub interaction sites in ARISC-RAP80, and they report effects of mutating or otherwise eliminating some of those sites on both in vitro DUB activity and recruitment to DNA damage foci. However, there is no quantitative assessment of the relative contributions of those sites. I consider this a major deficiency in an otherwise strong study. Such data would go far to dispel some uncertainties about how to interpret the results (e.g., see #3 below).

We thank the reviewer for these comments. To determine the contribution of each ubiquitin interacting site, we took advantage of our inactive ARISC(E33A)–RAP80 complex and introduced mutations or deletions in BRCC36 (S98K), Abraxas1 (Δ42-55), or BRCC45 (Δ161-170, referred to as ΔLoop) (**Fig. 4b**). We then tested the resulting variants in spectral shift assays (Dianthus, Nanotemper) against K63-linked Ub6 chains. ARISC(E33A)–RAP80 bound to K63-Ub6 with moderate affinity, which was strongly reduced in the presence of the ARISC(E33A)$^{BRCC36(S98K)}$–RAP80 and ARISC(E33A)$^{Abraxas1(\Delta 42\text{-}55)}$–RAP80 mutants (**Fig. 4c**). Deletion of BRCC45 residues 161-170 also reduced ARISC(E33A)–RAP80 binding to K63-Ub6, albeit to a lesser extent compared to the BRCC36 and Abraxas1 variants (**Fig. 4c**). These data are consistent with our enzymatic assays and structural analyses (**Fig. 2a**, **Fig. 3d,f,i,j**, **Fig. 4a,f**, **Fig. 5a,b**, **Supplementary Fig. 9b**, and **Supplementary Fig. 10b**), and suggest prominent roles for BRCC36 and Abraxas1 in supporting ARISC–RAP80 binding to ubiquitin chains. Our results also indicate that the BRCC45 UEV-N loop region (residues 161-170) likely mediate some contacts with ubiquitin chains, in agreement with our positioning of Ub (P4) and earlier cross linking-mass spectrometry analyses (**Fig. 2d-h**, **Fig. 5f-i**, **Supplementary Fig. 5c,d,g**, **Supplementary Fig. 10a**, and **Supplementary Fig. 11c,d,f**).

3. That inactive BRCC36 (E33A) showed significantly reduced (ARISC-RAP80) recruitment to IRIF damage foci (Fig. 3g; Extended Fig. 5c) was attributed (line 503) to reduced polyUb binding. Given that there are several other interactions that can engage K63-polyUb, that is quite surprising. To support the authors' contention, in vitro binding (point #2) would be very useful. Another possibility that should be explored is that the ARISC mutations that reduced ARISC-RAP80 at IRIF had their effects by lowering levels of assembled ARISC-RAP80 rather than through reduced polyUb binding. Experiments to evaluate levels of the ARISC-RAP80 complex would address this. Some characterization of RAP80, MERIT40 and BRCC45 levels in BRCC36 E33A knock-in cell lines were reported in Jiang et al. (JCB, 2022), but the effect on the assembled complex is unclear.

We thank the reviewer for these observations and we agree that presence of multiple ubiquitin binding sites within the complex makes it hard to observe differences in recruitment to damage foci when a single site is mutated.

We previously found that ARISC(E33A)–RAP80 is hyper-ubiquitylated in cells, and the loss of complex recruitment is a result of the RAP80 UIMs being occupied by ubiquitin chains in the DUB-inactive complex[3]. Similarly, mutants that cannot productively bind and cleave chains would be expected to have a reduction in IRIF levels as observed for the BRCC36(S98K) mutant (**Fig. 4d,e**, previously Fig. 3g and Extended Data Fig. 5c).

We now clarify this point in the main text, referencing our earlier studies: *"These data therefore indicate that BRCC36-mediated recruitment to ubiquitylated chromatin is lost in the auto-inhibited E33A variant[40], and in the BRCC36 S98K mutant where engagement with ubiquitin chains in its active site is impaired."*

Regarding the complex assembly of the mutants, we were able to purify stoichiometric amounts of each mutant after two affinity and one size-exclusion chromatography steps (**Fig. 4b** and **Supplementary Fig. 9a**), suggesting complex assembly is robust even after three rigorous purification steps. This is also consistent with the surface-exposed nature and non-structural role of these residues.

4. The results from comparison of linear and cyclic K63-polyUb are a bit confusing and not wholly convincing. First and foremost, gel-based evidence that cycK63-polyUb chains are cleaved more efficiently than the linK63-polyUb counterparts warrant quantitation and evidence of reproducibility. Second, the authors apparently did not consider that the activity against the cyclic chains might be even greater than it appears in the gels; whereas a single cut in a linear substrate would generate smaller (poly)Ub fragments, reducing the size of a cyclic chain would require two cuts. Third, the authors might consider that a reason cyclic chain could be preferred is that, unlike with linear polyUb, whichever Ub units occupy the P1 and P1' sites all the remaining Ub distal and proximal to the cleavage site will be in the same register. Fourth, the distal-end preference for cleavage by the ARISC DUB (p. 24) seems inconsistent with the claimed preference for cyclic chains (which necessarily undergo endo-cleavage). Finally, the authors speculate (line 892) about the physiological function of the cycK63-polyUb; they should at least acknowledge that such chains may be an artifact of their in vitro chain synthesis reaction and are inconsequential in cells.

We thank the reviewer for pointing this out. We have repeated these assays and carefully normalised input levels. Results using silver staining showed a higher activity of ARISC and ARISC–RAP80 towards linear K63-Ub5 chains compared to cyclical substrates when inputs are normalised (**Figure R2a**). To further clarify this aspect, we repeated the same assays with Oriole staining, using two concentrations of our DUB complexes (5 nM and 10 nM) and a higher concentration of K63-Ub5 chains (2 μM). We again detected higher activity of ARISC and ARISC–RAP80 against linear K63-Ub5 chains compared to their cyclical counterpart (**Figure R2**). We concluded that ARISC and ARISC–RAP80 display moderately higher activity towards linear Ub5 chains compared to cyclical substrates and agree with the reviewer that cyclical chains would require an extra cut. We have included the data obtained with Oriole staining in **Fig. 1g** of the revised manuscript, and commented on this aspect in page 8, lines 241-244.

As suggested by the reviewer, we now acknowledge that our cyclical chains might be an artifact of the *in vitro* system in the Discussion section (see page 32, lines 966-968).

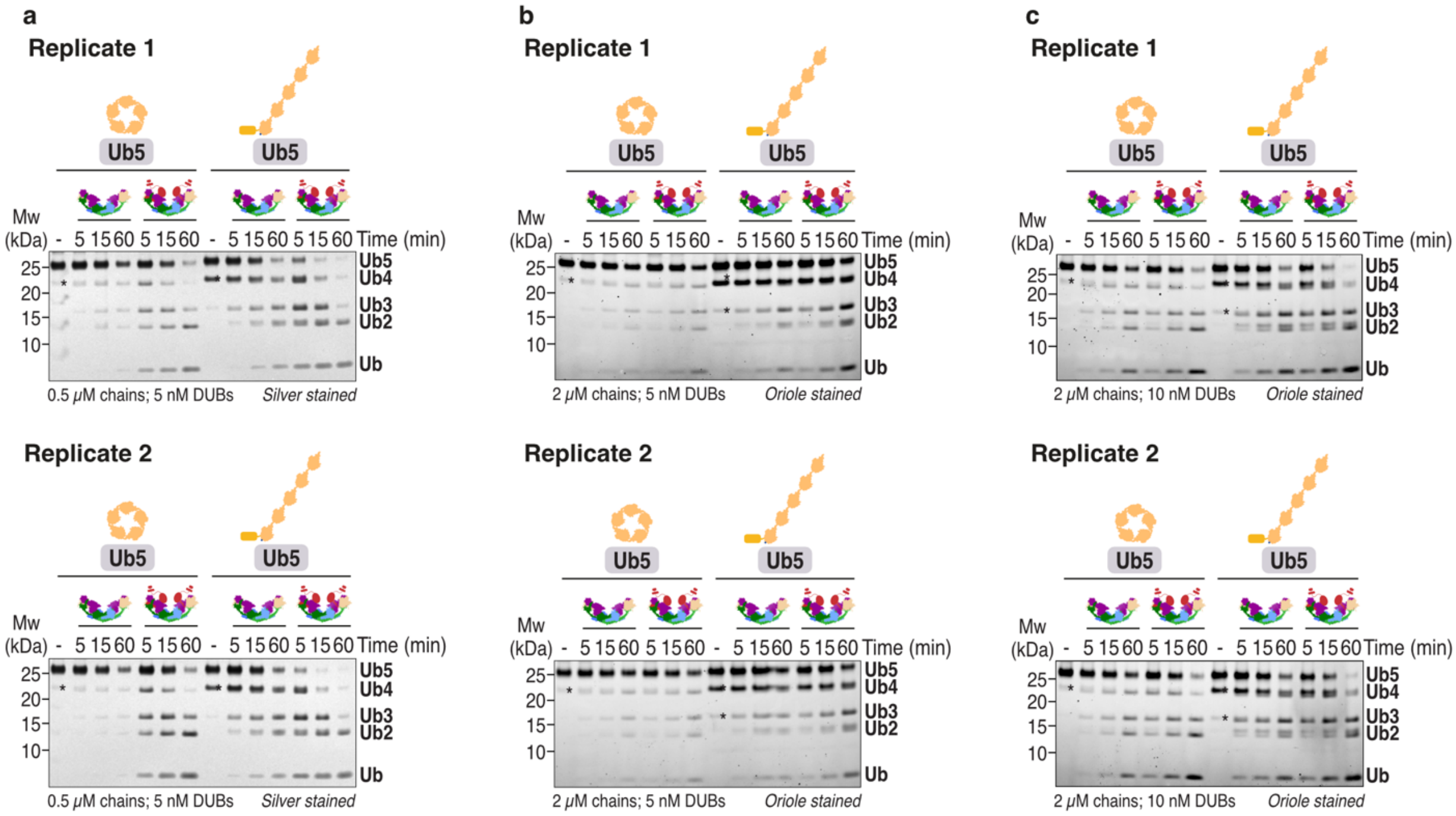

Figure R2. Comparison of ARISC and ARISC–RAP80 enzymatic activity against cyclical and linear K63-Ub5 chains.
a, Gel-based ubiquitin chain cleavage assays. Cyclical and linear K63-Ub5 chains (0.5 µM) were incubated with ARISC or ARISC–RAP80 (5 nM) for the indicated time points. Cleavage activity was analysed by SDS-PAGE and silver staining. Data corresponding to two independent experiments are shown. **b,** As in **a**, but in the presence of 2 µM cyclical and linear K63-Ub5 chains. Cleavage activity was analysed by SDS-PAGE and oriole staining. Data corresponding to two independent experiments are shown. **c**, As in **b**, but in the presence of 10 nM ARISC and ARISC–RAP80 complexes. Data corresponding to two independent experiments are shown. Ub, ubiquitin; DUB, deubiquitylating enzyme. * indicates lower molecular weight ubiquitin species.

5. ARISC DUB activity was reduced when N-terminal MERIT40 was deleted (ARISCΔN), but little effect was observed with deletion of C-terminal Abraxas1 (ARISCΔC) or N-terminal MERIT40 + C-terminal Abraxas (ARISCΔNΔC). Shouldn't (ARISCΔNΔC) affect catalytically activity similarly to ARISCΔN?

We thank the reviewer for highlighting this aspect. Although ARISCΔN and ARISCΔNΔC should, in principle, display very similar enzymatic activities, it is possible that concomitant deletions of the MERIT40 and Abraxas1 N- and C-termini causes conformational rearrangements within ARISC that could compensate for the loss of these regions. In our unpublished work we have noticed that ARISCΔNΔC is similar in structure to BRISC (which is usually ~10x more active), possibly accounting for the results seen here, although this needs further evaluation.

6. Regarding BRCC45 UEV-C interactions with Ub seen in the cryo-EM structure and predicted by AlphaFold: does mutating BRCC45 UEV-C have any effect on BRCA1-A accumulation at IRIF?

To determine the relevance of the BRCC45 UEV-C:ubiquitin interactions for ARISC–RAP80 function in cells, we mutated amino acids in the BRCC45 UEV-C domain (**Fig. 7g**, previously Fig. 6f) and tested the resulting variants for their ability to form BRCC45 IRIF. Both BRCC45$^{S350Y+P351N+R352D}$ and BRCC45$^{W353A+R361A}$ did not cause any difference in IRIF levels when compared to wild type (WT) protein (**Supplementary Fig. 14e,f**), indicating that interactions between BRCC45 UEV-C and ubiquitin are not necessary for ARISC–RAP80 recruitment to damaged chromatin. We have included these results on pages 28 and 29 (lines 870-881).

The multivalent nature of the ubiquitin-interaction sites makes it difficult to obtain measurable results when mutating individual sites, and we have made this point in the revised manuscript.

7. (line 367) Given the prominent role played by RAP80 in polyUb binding and the increased activity of ARISC-RAP80 over ARISC, activity assays should include ARISC-RAP80 with the UIMs mutated to evaluate effects on longer-chain substrates. Along these lines, does addition of full-length RAP80 to ARISC skew the position of the first cleavage with long-chain substrates?

We thank the reviewer for highlighting this. In response to the reviewer's suggestion, and supported by our earlier cross linking-mass spectrometry analyses (**Fig. 2d-g**), we purified stoichiometric amounts of two ARISC–RAP80 variants in which the RAP80 UIM and ZnF domains were deleted (**Fig. 1a,e**). We then assayed these protein complexes, together with ARISC–RAP80, against our fluorescently-labelled Ub4 substrate. We detected a considerable reduction in ARISC–RAP80 ΔUIMs activity compared to WT complex, while the activity of ARISC–RAP80 ΔZnF was only marginally reduced (**Fig. 1f**). These results indicate that: 1) the UIM regions represent the major ubiquitin binding site in RAP80; and that 2) the ZnF region may assist in ubiquitin binding, albeit with reduced affinity.

Following the reviewer's suggestion, we performed additional activity assays where we added increasing concentrations of full-length (FL) RAP80 to ARISC. We did not observe any obvious differences in the first cleavage event under the conditions tested (**Figure R3**).

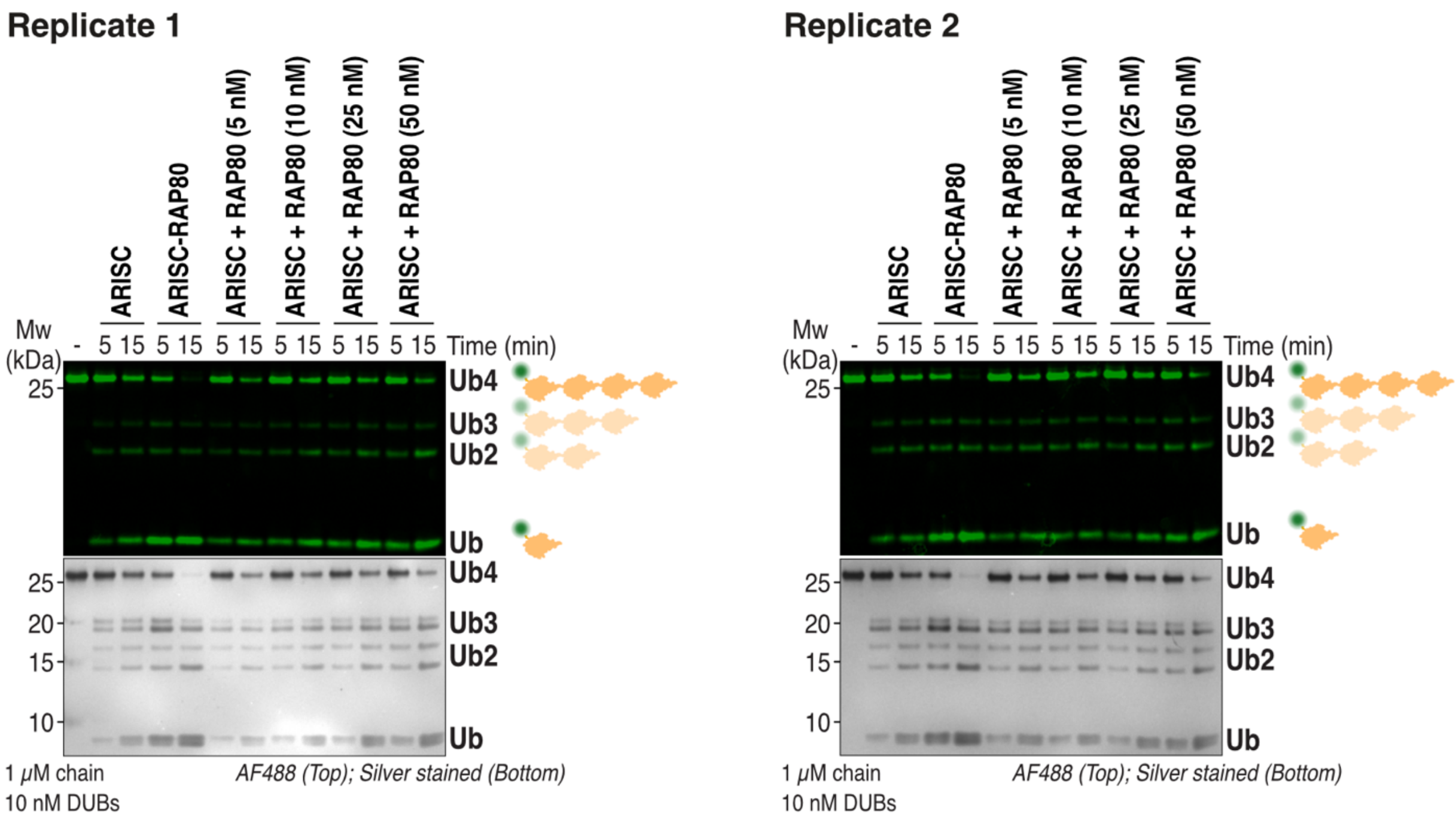

Figure R3. Addition of FL RAP80 to ARISC does not enhance ARISC enzymatic activity nor cause changes in ubiquitin chain cleavage pattern.

Gel-based ubiquitin chain cleavage assays. Alexa-Fluor (AF488) labelled distally (AF488-^{Cys}Ub4^{K63R}) blocked K63-Ub4 chains (1 μM) were incubated with ARISC or ARISC–RAP80 (10 nM) for 60 minutes. Parallel reactions containing ARISC (10 nM) and increasing concentrations of RAP80 (5 nM-50 nM) were also incubated in the presence of the same substrate and for the same length of time. Cleavage activity was analysed by SDS-PAGE; gels were subsequently scanned using an iBright FL 1500 gel imaging system (excitation: 455-485 nm, emission: 508-557 nm) prior to being silver stained. Data corresponding to two independent experiments are shown.

8. In the Fig. 4 legend (lines 691-694), the title says the cryo-EM structures are for ARISCΔC(E33A):K63-Ub7 and ARISCΔC(E33A):K63-Ub4 complexes, but the legends for 4a and 4b say ARISC(E33A)-RAP80:K63-Ub7 and ARISC(E33A)-RAP80:K63-Ub7

We thank the reviewer for highlighting this typo which we have corrected (**Fig. 5**, previously Fig. 4).

Reviewer #2 (Remarks to the Author):

In this manuscript, Foglizzo et al present a powerful structural and biochemical analysis of substrate recognition by ARISC-RAP80 (also known as BRCA1-A) metallo deubiquitinase (Dub) complex that regulates the recruitment of the larger BRCA1 supercomplex to ubiquitinated foci at damaged DNA sites and DSB repair within chromatin. Using enzymatically produced K63-linked polyubiquitin chains as substrates they managed to capture the structure of substrate bound state of the ARISC-RAP80 complex using single-particle cryo-EM at relatively high resolution. The structure allowed visualization of a larger piece of the polyubiquitin chain substrate bound to the complex revealing K63-isopeptide linked diubiquitin in the active site of the BRCC36 Dub catalytic unit in a productive orientation, with additional ubiquitins occupying previously unexpected sites on the complex beyond the Brcc36 catalytic unit.

The structural results were supported by solution crosslinking mass spectrometry, which identifies additional ubiquitin interaction sites distal to the catalytic core where ubiquitin binding contributes to catalytic efficiency. Guided by structure the authors created and validated mutants using both in vitro deubiquitinase assays and cell-based assays assessing BRCA1-A recruitment to DSBs. Overall, the study represents a significant mechanistic advance in understanding how a multi-protein nuclear DUB complex recognizes and processes polyubiquitin chains in the DNA damage response pathway. The cryo-EM work is rigorous with solid the structural interpretation, and the manuscript is clearly written with high-quality figures in line with expectations for a paper in Nature Communications.

Specific Comments:

(1) The authors have prepared a stochiometric complex of ARISC with full-length RAP80 and mixed it with cyclic Ub7 polymer for cryo-EM reconstruction. The cyclic substrate seems better than the corresponding linear polyubiquitin substrate, so it makes good sense to use it for cryo-EM studies. The observed ubiquitin groups at positions in BRCC36 active site (P1'-P1) align well with the expected positions of the same sites in AMSH-LP, a prototypical JAMM family Dub. Two additional ubiquitin densities (P3 and P4) mapped onto ABRAXAS1 with high confidence, and the isopeptide linkages connecting these positions are well resolved.

The occupancy at these sites and the novel arrangement of ubiquitin groups in the K63-chain provides interesting mechanistic insights. However, density corresponding to ubiquitin engagement at the BRCC45 UEV domains is absent, which could be perhaps due to lower affinity as these sites. It is plausible that the lack of ubiquitin density at the arms could be due to the constraints imposed by the non-native cyclic structure of the polyubiquitin chain. It would strengthen the conclusions if the authors included crosslinking mass spectrometry data collected using linear Ub7 chain (as an additional panel in Figure 2). With the long linear substrate like Ub7 there could be more crosslinks between ubiquitin and BRCC45 domains than what has been observed in Figure 2g. Please note that going from Ub4 (linear) to Ub7 (cyclic) there are hardly any more crosslinks from Ub to BRCC45 UEV domains (if not fewer) which does correlate with difference in catalytic activity between these substrates.

We thank the reviewer for these observations. We performed parallel cross-linking mass spectrometry analyses of ARISC(E33A)–RAP80 with cyclical and linear K63-Ub7 chains, and included these new data in **Fig. 2g,h** of the revised manuscript. We did not observe any differences in the number and/or site of cross-links between BRCC45 and cyclical or linear Ub7 substrates. Similarly, we detected comparable cross-links between Abraxas1 or RAP80 and cyclical or linear K63-Ub7 chains.

Our data therefore indicate that ARISC–RAP80 adopts a conserved mode of interaction with both cyclical and linear ubiquitin substrates (especially when chains are long), whereby shared ubiquitin-interacting sites in ARISC–RAP80 subunits mediate substrate recognition and promote downstream ubiquitin chain cleavage.

(2) The nature of cyclic structure has not been completely established. The loss of water (dehydration) is taken as evidence for cyclic structure, which is reasonable but not conclusive. They argue unanchored Lys63 linked chains have a propensity to form head-to-tail cyclic conformation bringing Lys63 of the first and G76 carboxylate of the last ubiquitin to proximity to be linked by the E2 enzyme. In this unexpected reaction, it is possible that the Met 1 alpha-amino group of the first Ub becomes linked to the last Gly76. Indeed, to prevent cyclization they introduce two modifications (blocking the N-terminus and mutating K63 to R). Since cryo-EM did not reveal the complete structure of the substrate, it is important to consider a different way of establishing the nature of the cyclic structure of the polyubiquitin chain substrate.

We thank the reviewer for highlighting this possibility. To better assess the nature of our cyclical polyubiquitin chains, we assayed ARISC, ARISC–RAP80, AMSH* (a STAM2–AMSH fusion with selectivity for K63-linked ubiquitin chains)[4-6], and OTULIN (an OTU family member with exclusive specificity for Met1-linked chains)[7,8] against cyclical K63-Ub5 and linear Met1-Ub4 chains (**Supplementary Fig. 4a,b**). We observed robust activity of ARISC, ARISC–RAP80, and AMSH* towards cyclical K63-Ub5 chains, with full conversion of the Ub5 parent band to monoubiquitin. Similarly, we detected high activity of OTULIN in the presence of Met1-Ub4 chains (**Supplementary Fig. 4b-d**). Most importantly, ARISC, ARISC–RAP80, and AMSH* did not display any activity towards Met1-Ub4, which is consistent with established literature. Likewise, OTULIN did not hydrolyse our cyclical K63-Ub5 substrate (**Supplementary Fig. 4b-d**) since non-cyclic and cyclic chains are known to have different electrophoretic mobility[9].

Collectively, these data demonstrate that cyclisation of our ubiquitin chains does not involve Met1 linkages nor other linkage types mediated by additional lysine residues in ubiquitin.

(3) The authors should consider validating P2 and P3 interfaces observed in their structure through the cell based IRIF assay (such as mutant of Abraxas1 E4).

We thank the reviewer for this suggestion. However, with the exception of Abraxas1 D206 contacting E64 in Ub (P2), we do not observe any other main- or side-chain interactions between ARISC and this ubiquitin molecule (**Figure R4a**). Furthermore, we have already deleted amino acids 42-55 in Abraxas1, which directly interact with Ub (P3), and have shown that these residues, although required for ARISC–RAP80 interaction *in vitro*, do not affect DUB recruitment to DNA damage sites when deleted (**Fig. 3f,i,j**, **Fig. 4c,f**, and **Supplementary Fig. 9g,h**).

Of note, the N-terminus of Abraxas1 and residues 42-55 are also in proximity to the isoleucine 44 acidic patch in ubiquitin, one of the most important sites for ubiquitin interaction with its binding partners (**Figure R4b** and **Fig. 3g**)[10].

Collectively, these data and the multivalent nature of ubiquitin recognition suggest that single mutations of additional Abraxas1 residues are unlikely to impact ARISC–RAP80 recruitment to damage foci in cells.

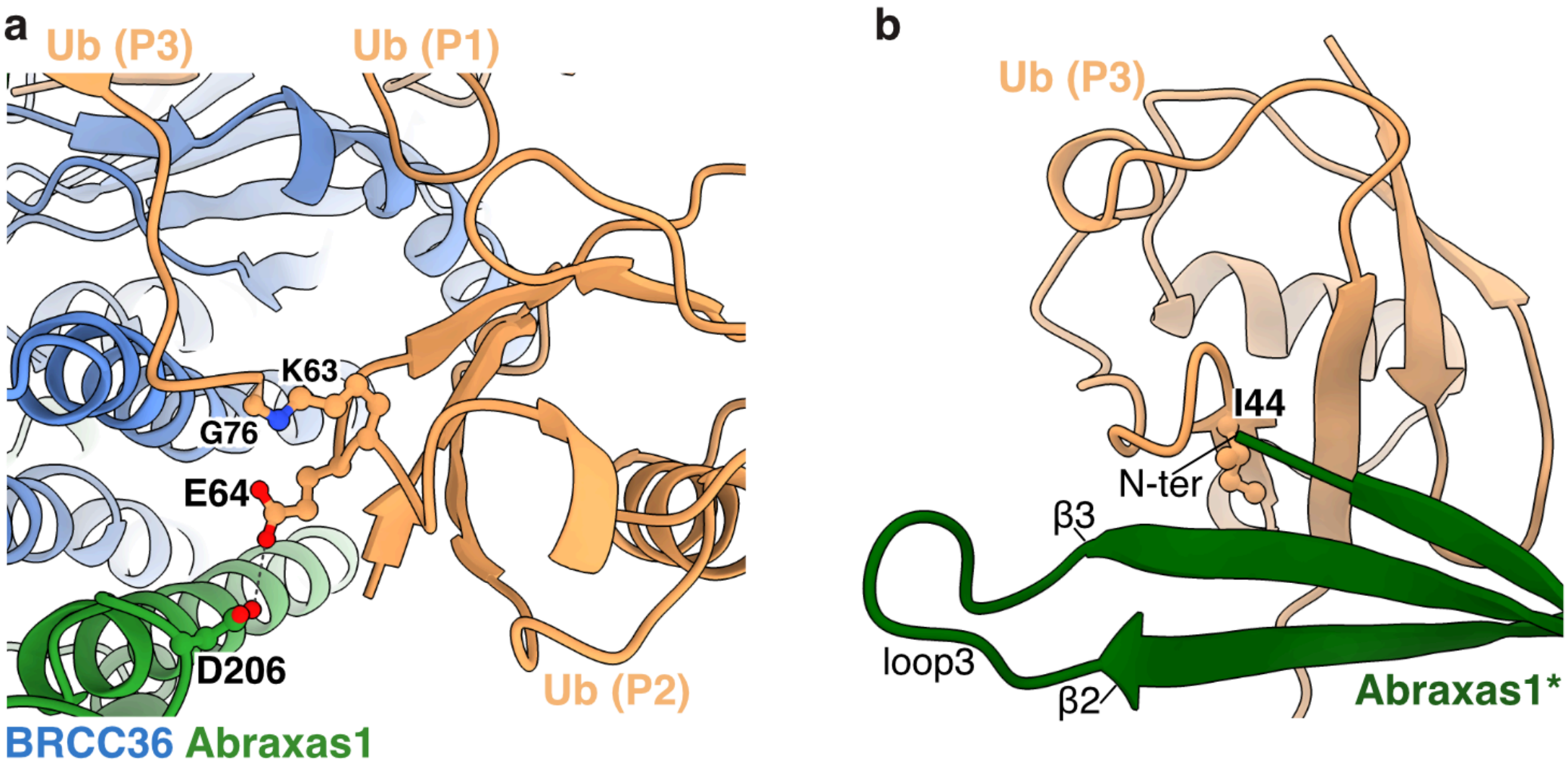

Figure R4. Structural analyses of the BRCC36:Abraxas1:Ub (P2) and Abraxas1*:Ub (P3) interfaces. **a,b,** Close-up views and structural details of the BRCC36:Abraxas1:Ub (P2) (**a**) and Abraxas1*:Ub (P3) (**b**) interfaces. ARISC subunits and ubiquitin molecules are shown as cartoon, and colored light and dark green (Abraxas1 and Abraxas1*), light blue (BRCC36), and light orange (ubiquitin). Interacting residues in Abraxas1 and Ub (P2) or Ub (P3) are shown as ball & sticks; direct contacts between amino acids are indicated with dashed black lines.

(4) In the model presented in Figure 6g, would it not make more sense to connect the ubiquitin chain at the catalytic unit to that emanating from the nucleosome?

We thank the reviewer for this suggestion. We have now revised our final model to address this point (**Fig. 8**).

(5) The authors need to explain the mechanistic implication of their observation of only one of BRCC36 being occupied by ubiquitin. Does this asymmetry imply any functional relevance?

We thank the reviewer for underlying this aspect and have expanded on this point in the revised version of our manuscript.

Overlaying the ubiquitin chains onto the unbound BRCC36* active site produces only a small clash between the α1 C-terminal regions of the two Ub (P1′) molecules. We have now better illustrated this in **Supplementary Fig. 5e**, adding a top view of the ARISC–RAP80 complex showing that all ubiquitin moieties except Ub (P1′) can be accommodated on both BRCC36 active sites (*left*). We also show a zoomed-in view of the small overlap between the two Ub (P1′) molecules (*right*).

Based on our analyses, we believe that structural re-arrangements or re-positioning of the ubiquitin moieties within ARISC may allow both BRCC36 active sites to concomitantly engage with their ubiquitin substrates. Alternatively, it is possible that both chains are bound simultaneously but cleavage happens in alternating cycles, thus accommodating only one Ub (P1’) at a time. We have included these options in the Discussion section of the revised manuscript (see page 33, lines 996-1003).

Reviewer #4 (Remarks to the Author):

The manuscript from Foglizzo and colleagues entitled "Mechanism of K63-linked polyubiquitin recognition and cleavage by the BRCA1-A complex" described the structural and biochemical characterization of a deubiquitinase complex comprised of ARISC and RAP80 in complex with K63-linked polyubiquitin chains of different lengths. The ARISC complex functions in DNA damage signaling. This portion of this complex is itself comprised of protein subunits including Abraxis1, BRCC36, BRCC45 and MERIT40. The data show how ARISC-RAP80 engage K63 linked polyUb chains in a manner distinct from previous K63 interacting proteins, and perform appropriate follow up studies through mutagenesis of key residue to validate the conclusions biochemically and in cells. The authors take the data one step further and elucidate how these interactions between the DUB complex and K63 linked poly Ub are structurally and kinetically modulated by the length and configuration of the chain (linear vs circular). Finally, they conclude the paper by demonstrating the catalytic mechanism for the DUB activity, which acts on the distal Ub subunit within the K63 polyUB chain. Overall, the data in this paper are impressive and the interpretation of the results consistent with the observations. The work represents an important advance for the ubiquitin and DNA damage field. Below are a number of questions that arose during review of the manuscript that require clarification and/or suggestions for improving the impact of this work.

For the DUB catalytic assays in Fig 1c-e, the figure legend indicates that n=3 data were collected. I find the data very informative as is. That said, it would be additionally informative if certain aspects of the data were presented in a quantitative manner, whether it be for loss of the full length parental K63 chain, or formation of partially or completely disassembled Ub products of the assay.

We thank the reviewer for highlighting this point. As discussed in response to Reviewer #1 (comment 1, pages 2 and 3), we used silver staining to visualise our gel-based cleavage assays. Despite its high sensitivity, this stain is characterised by a fast band saturation and a narrow dynamic range, and therefore is not fully reliable for band quantification[1,2]. However, we have repeated our assays using fluorescently labelled poly-Ub substrates. These assays and the related quantifications can be found in **Fig. 1f**, **Fig. 6a,b**, and **Supplementary Fig. 1d,e**.

Looking carefully at the data in Fig 1e, it appears that linear Ub5 chains are not fully processed (upper band eliminated), while the circular form appears to be more extensively processed. This surprised me given that the paper argues that circular Ub5-K63 chains were in fact refractory to cleavage as compared to linear chains or circular chains with additional subunits. What is the explanation for this?

We apologise for the confusion. As already mentioned in response to Reviewer #1 (comment 4, pages 4 and 5), we have repeated these assays multiple times varying the concentrations of our DUB complexes and chains as well as by using silver and Oriole staining (**Figure R2**). We consistently detected higher activity of ARISC and ARISC–RAP80 against linear K63-Ub5 chains compared to their cyclical counterpart, especially after carefully normalising the input lanes (**Figure R2**).

We concluded that ARISC and ARISC–RAP80 display moderately higher activity towards linear Ub5 chains compared to cyclical substrates (**Fig. 1g** of the revised manuscript), and commented on this aspect in page 8, lines 241-244.

Following on this point, it was not clearly described how the 6x-His and K63R mutated proteins, added to chain formation assays substoichiometrically, would yield different reaction products. Specifically, I could not discern how K63R mutant Ub could alter cyclization when added to a distal position. Taking this one step further, the authors provided circumstantial evidence of chain cyclization (through intact mass analysis), but I did not see

any irrefutable direct evidence to show precisely the precise nature of the proposed cyclization. The authors should provide a more thorough description of what they do and do not know about the proposed cyclized chains being used in the assays, and if possible, generate data that directly demonstrates the position of cyclization. Is it possible that the cyclized chains represent a mixed population of differentially linked products?

We assembled distally blocked K63-linked ubiquitin chains in a manner similar to the protocol described in Martinez-Fonts et al. (2016)[11], and have provided a detailed explanation of how these chains were assembled in the Methods section (see pages 39 and 40, lines 1232-1253). To further clarify the methodology, we have included a schematic in **Figure R5** below.

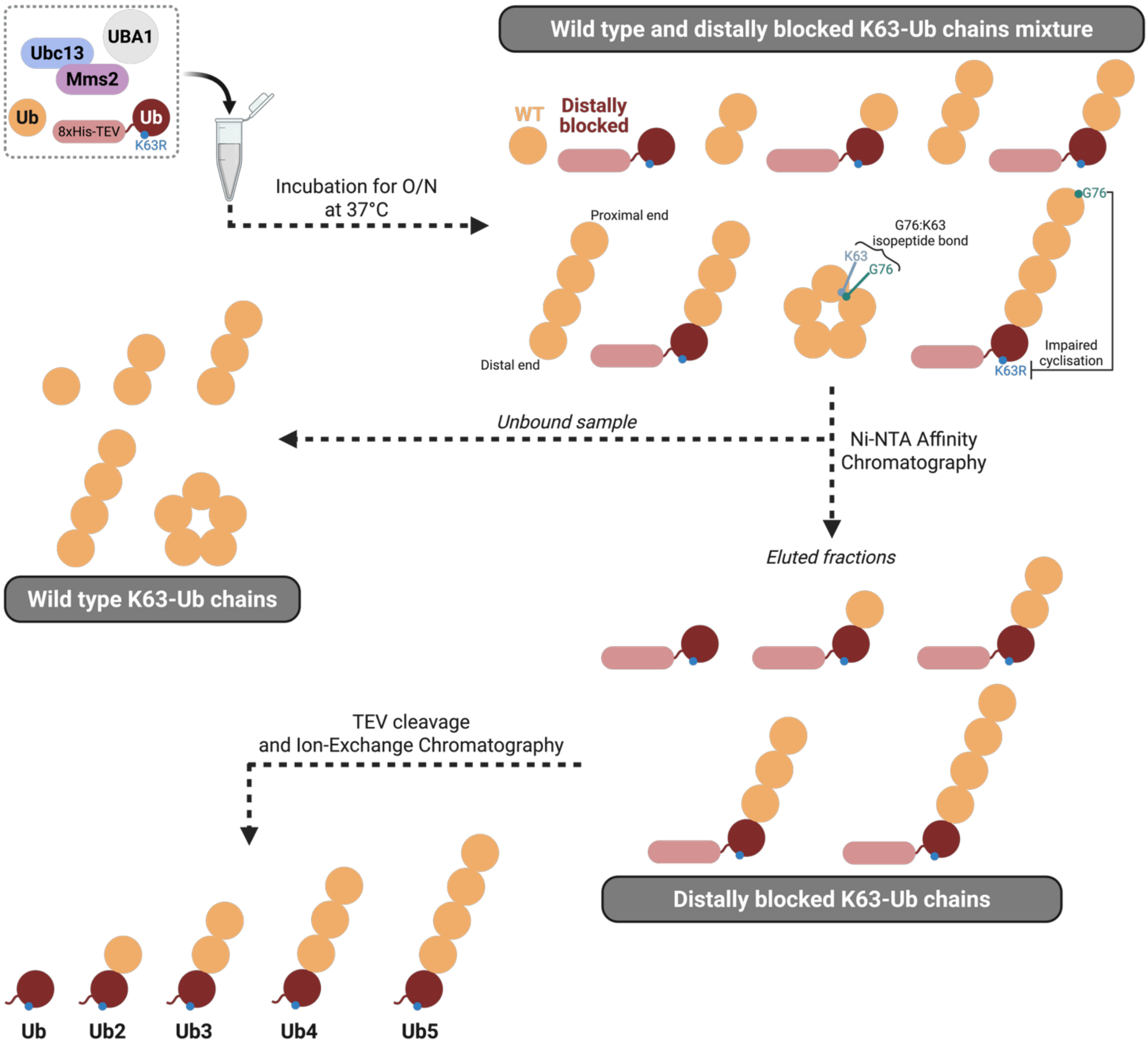

Figure R5. Enzymatic assembly and purification of distally blocked K63-Ub chains.
Schematic of the methodology employed to assemble and purify distally blocked K63-linked ubiquitin chains of different lengths. [Editorial note: created in BioRender; Foglizzo, M. (2026) https://BioRender.com/7ymosu8.]

As it concerns the cyclisation of our K63-Ub5 and longer chains, we now provide a follow-up analysis discerning the nature of these substrates (see also response to Reviewer #2, comment 2, page 8). Our data, using selective DUB cleavage assays, demonstrate that cyclisation of our ubiquitin chains is through K63 and does not involve Met1 linkages or other linkage types. These results therefore support our initial hypothesis of a cyclic conformation,

whereby G76 of the distal ubiquitin forms an isopeptide bond with K63 of the proximal ubiquitin moiety.

The data describe a novel Ub1-Ub3 interaction in the bent K63 polyUb chains engaged with ARISC-RAP80. Have the authors tested mutant forms of K63 polyUb that would not permit these interactions (e.g. text mentions positions the I44 hydrophobic patch and D58 acidic residue) to see whether these residues/interactions are required for K63-polyUb cleavage by ARISC-RAP80?

We thank the reviewer for highlighting this. However, mutations of the I44 and I36 hydrophobic patches as well as of the D58 acidic residue in ubiquitin have been shown to significantly impair ubiquitin chain synthesis and/or correct recognition of polyubiquitin chains by deubiquitylating enzymes and other ubiquitin-binding proteins[10,12-15], making the generation of reagents for our experiments nearly impossible and downstream results difficult to interpret.

We have already validated polyubiquitin chain engagement by ARISC–RAP80 via biochemical and cell-based approaches, and have included additional enzymology and biophysical analyses (using structure-guided mutants) in the revised version of the manuscript (see **Fig. 1e,f**, **Fig. 4**, **Supplementary Fig. 1d,e**, **Supplementary Fig. 9a,b,g,h**, and **Supplementary Fig. 10**). Collectively, these data provide strong evidence in support of our ARISC–RAP80 structural model.

Have the authors tested whether linear polyUb chains linked through M1 will biochemically engage the ARISC-RAP80 complex. If these chains are modeled into the same position at the K63 chains tested here, what factors make them incompatible for engagement/proteolysis.

We thank the reviewer for this comment. We have tested a similar hypothesis in response to Reviewer #2 (comment 2, page 8). As described in comment 3 above, our data suggest that ARISC–RAP80 does not cleave Met1-linked ubiquitin chains, even when tested for long times (up to 22 h) (**Supplementary Fig. 4**). This strongly suggests that ARISC does not productively engage Met1-linked chains, which is consistent with published literature.

Is their a structural or biochemical reason to explain why no chemical crosslinks observed between BRCC36 and K63-poly Ub chains?

We thank the reviewer for highlighting this point. We did observe cross-links between BRCC36 and ubiquitin chains within ARISC(E33A)–RAP80 and ARISCΔC(E33A) complexes, but most of them were not consistent between the two experimental replicates we originally carried out. The exceptions were BRCC36 cross-link sites between ARISCΔC(E33A) and linear K63-Ub4 or cyclical K63-Ub7, which were included in the original manuscript (**Fig. 5g,i** and **Supplementary Fig. 11f**, previously Fig. 4g,i and Extended Data Fig. 7f).

In the interim, we performed additional cross-linking mass spectrometry analyses of ARISC(E33A)–RAP80 with cyclical and linear K63-Ub7 substrates. We now consistently observe cross-links between BRCC36 and both ubiquitin chain types, which we show in **Fig. 2g,h**. Of note, most of the lysine residues in BRCC36 are buried within the BRCC36-Abraxas1 heterodimer or located in flexible loop regions, possibly hindering cross-linking efficiency.

The authors should offer additional text to describe what the P1' ubiquitin is. DO they believe that a secondary K63 polyUb chain engages with a catalytically active ARISC-RAP80 complex as part of its normal function?

We thank the reviewer for highlighting these aspects. We have now included additional text to define the different ubiquitin molecules in our polyubiquitin chains, and have clarified what the distal (P) and proximal (P') ubiquitin moieties are (see page 11, lines 319-322).

In regard to whether both ARISC–RAP80 catalytic sites can engage with K63-linked ubiquitin chains, we also addressed this aspect in response to Reviewer #2 (comment 5, page 9). We have included additional text (see page 33, lines 992-999) and a revised figure (**Supplementary Fig. 5e**, previously Extended Data Fig. 2e).

In Fig 3g, the authors test whether mutation of E33A and S98K of BRCC36 affect the formation of g-H2AX foci 4 hr post DNA damage through gamma irradiation. What additional mutations have been tested and do any show inhibition/enhancement of DUB activity? Text later in the paper speaks to BRCC45 residues including K270, K360 and K345, for example. The fact that these potentially engage with unbranched lysine residues on Ub itself if highly interesting.

We thank the reviewer for this comment. We also tested two Abraxas1 deletion constructs (Δ42-55 and Δ46-50) for their ability to affect IRIF formation, and have included these data in **Supplementary Fig. 9g,h**. As it concerns residues at the BRCC45 UEV-C:ubiquitin interface, we have also performed these experiments (see response to Reviewer #1; comment 6, pages 5 and 6). We have included these new data and discuss them accordingly (pages 28 and 29, lines 870-881; **Supplementary Fig. 14e,f**).

As the authors were attempting to elucidate DUB catalytic mechanism versus the distal or proximal chain subunit, and given the differences between linear chains and the purported circular chains, I could not help but wonder if the paper would benefit from appending the K63-linked polyUb chain onto a substrate like peptide or protein to show the way that the ARISC-RAP80 complex orients a non-polyUb feature in relation to the chain itself. While this may be more work than is reasonable for this paper, it would be highly informative for the field.

We thank the reviewer for this comment. While we agree that the suggested experiments would be highly informative for the field, we feel these are out of scope for the current manuscript as they would require significant efforts to generate new reagents and another cryo-EM structure, including subsequent validation work.

References

1. Protein Detection on Gels, Blots and Arrays – Section 9.3. *ThermoFisher Scientific* https://www.thermofisher.com/uk/en/home/references/molecular-probes-the-handbook/protein-detection-and-proteomics-technology/detection-of-the-total-protein-profile-in-gels-on-blots-on-microarrays-and-in-capillary-electrophoresis.html
2. White I.R., Pickford R., Wood J., Skehel J.M., Gangadharan B. & Cutler P. (2004) A statistical comparison of silver and SYPRO Ruby staining for proteomic analysis. *Electrophoresis*. 25(17):3048-3054. doi: 10.1002/elps.200405947
3. Jiang Q., Foglizzo M., Morozov Y.I., Yang X., Datta A., Tian L., Thada V., Li W., Zeqiraj E. & Greenberg R.A. (2022) Autologous K63 deubiquitylation within the BRCA1-A complex licenses DNA damage recognition. *J Cell Biol*. 221(9):e2021111050. doi: 10.1083/jcb.202111050

4. McCullough J., Clague M.J. & Urbé S. (2004) AMSH is an endosome-associated ubiquitin isopeptidase. *J Cell Biol*. 166(4):487-492. doi: 10.1083/jcb.200401141

5. Sato Y., Yoshikawa A., Yamagata A., Mimura H., Yamashita M., Ookata K., Nureki O., Iwai K., Komada M. & Fukai S. (2008) Structural basis for specific cleavage of Lys 63-linked polyubiquitin chains. *Nature*. 455(7211):358-362. doi: 10.1038/nature07254

6. Michael M.A., Elliott P.R., Swatek K.N., Simicek M., Pruneda J.N., Wagstaff J.L., Freund S.M.V. & Komander D. (2015) Assembly and specific recognition of k29- and k33-linked polyubiquitin. *Mol Cell*. 58(1):95-109. doi: 10.1016/j.molcel.2015.01.042

7. Keusekotten K., Elliott P.R., Glockner L., Fiil B.K., Damgaard R.B., Kulathu Y., Wauer T., Hospenthal M.K., Gyrd-Hansen M., Krappmann D., Hofmann K. & Komander D. (2013) OTULIN antagonizes LUBAC signaling by specifically hydrolyzing Met1-linked polyubiquitin. *Cell*. 153(6):1312-1326. doi: 10.1016/j.cell.2013.05.014

8. Rivkin E., Almeida S.M., Ceccarelli D.F., Juang Y.-C., MacLean T.A., Srikumar T., Huang H., Dunham W.H., Fukumura R., Xie G., Gondo Y., Raught B., Gingras A.-C., Sicheri F. & Cordes S.P. (2013) The linear ubiquitin-specific deubiquitinase gumby regulates angiogenesis. *Nature*. 498(7454):318-324. doi: 10.1038/nature12296

9. Yao T. & Cohen R.E. (2000) Cyclization of polyubiquitin by the E2-25K ubiquitin conjugating enzyme. *J Biol Chem*. 275(47):36862-36868. doi: 10.1074/jbc.M006050200

10. Agrata R. & Komander D. (2025) Ubiquitin – A structural perspective. *Mol Cell*. 85(2):323-346. doi: 10.1016/j.molcel.2024.12.015

11. Martinez-Fonts K. & Matouschek A. (2016) A rapid and Versatile Method for Generating Proteins with Defined Ubiquitin Chains. *Biochemistry*. 55(12):1898-1908. doi: 10.1021/acs.biochem.5b01310

12. Saha A., Lewis S., Kleiger G., Kuhlman B. & Deshaies R.J. (2011) Essential role for ubiquitin-ubiquitin-conjugating enzyme interaction in ubiquitin discharge from Cdc34 to substrate. *Mol Cell*. 42(1):75-83. doi: 10.1016/j.molcel.2011.03.016

13. Roscoe B.P. & Bolon D.N.A. (2014) Systematic exploration of ubiquitin sequence, E1 activation efficiency, and experimental fitness in yeast. *J Mol Biol*. 426(15):2854-2870. doi: 10.1016/j.jmb.2014.05.019

14. Scott D., Oldham N.J., Strachan J., Searle M.S. & Layfield R. (2015) Ubiquitin-binding domains: mechanisms of ubiquitin recognition and use as tools to investigate ubiquitin-modified proteomes. *Proteomics*. 15(5-6):844-861. doi: 10.1002/pmic.201400341

15. Deol K.K., Lorenz S. & Strieter E.R. (2019) Enzymatic Logic of Ubiquitin Chain Assembly. *Front Physiol*. 5:10:835. doi: 10.3389/fphys.2019.00835
