# Supplementary material for: Mechanism of K63-linked polyubiquitin recognition and cleavage by the BRCA1-A complex
Source: Nat Commun. 2026 Aug 11;17:8091. doi: 10.1038/s41467-026-75795-y (PMC13462971; doi:10.1038/s41467-026-75795-y)

Source Data Fig. 4

Fig. 4a (*Ub2*)

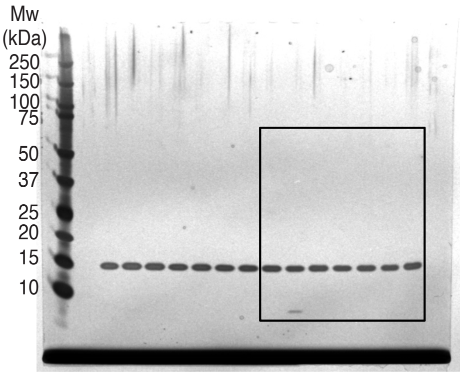

Fig. 4a (*Ub4*)

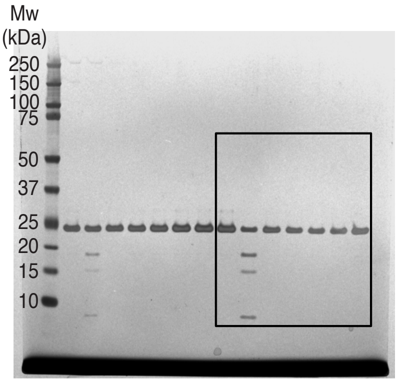

Fig. 4a (*Ub7*)

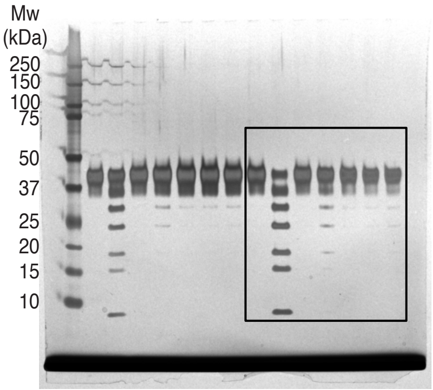

Fig. 4b

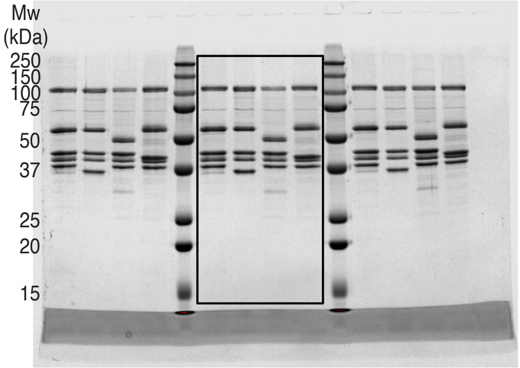

Fig. 4f (*15 minutes time point*)

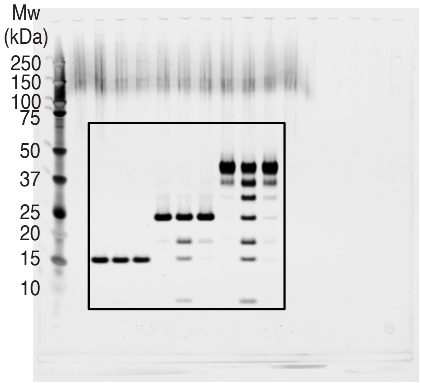

Fig. 4e (*left*)

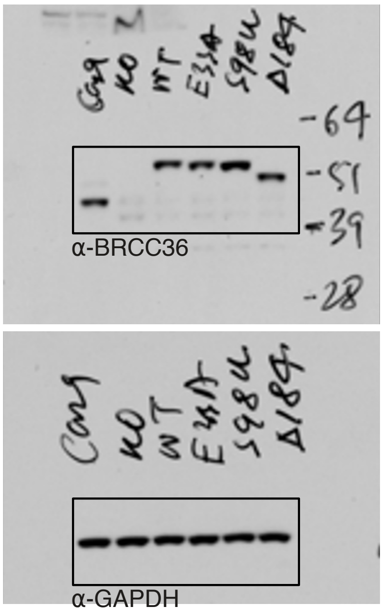

Fig. 4f (*60 minutes time point*)

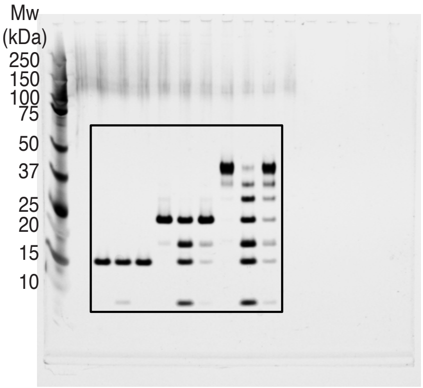

Supplement: Supplementary file 4 [file 41467_2026_75795_MOESM4_ESM.zip › Source Data/Source Data Figure 4.pdf]
