# Supplementary material for: Mechanism of K63-linked polyubiquitin recognition and cleavage by the BRCA1-A complex
Source: Nat Commun. 2026 Aug 11;17:8091. doi: 10.1038/s41467-026-75795-y (PMC13462971; doi:10.1038/s41467-026-75795-y)

Source Data Fig. 1

Fig. 1b

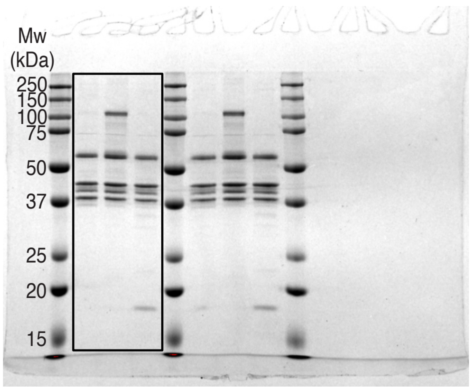

Fig. 1c (*Ub2 and Ub3*)

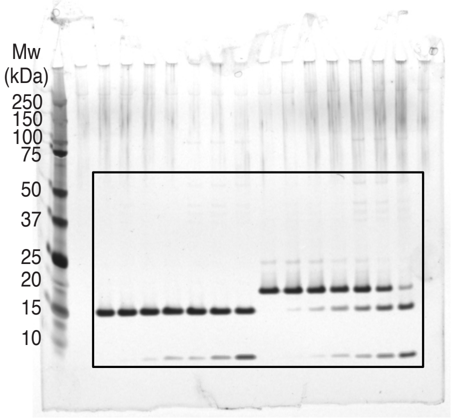

Fig. 1c (*Ub4 and Ub5*)

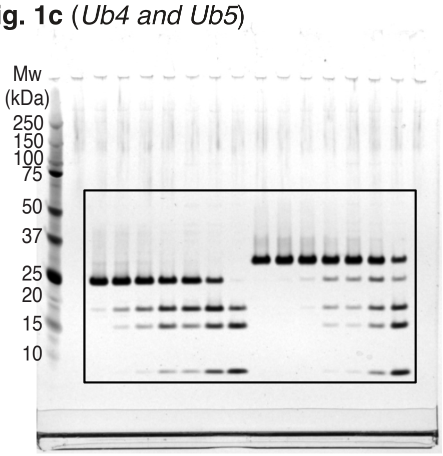

Fig. 1c (*Ub6 and Ub7*)

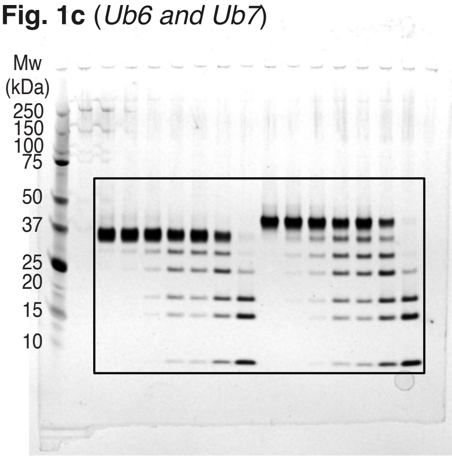

Fig. 1c (*Ub8 and Ub9*)

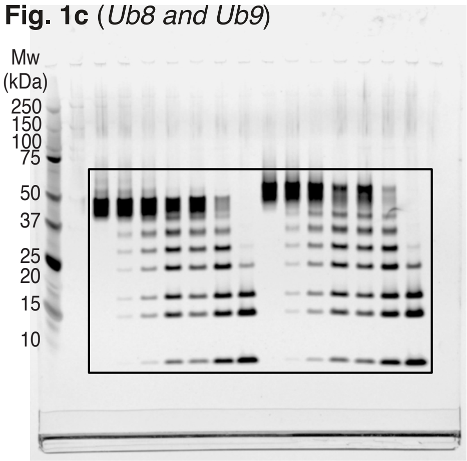

Fig. 1d (*Ub2*)

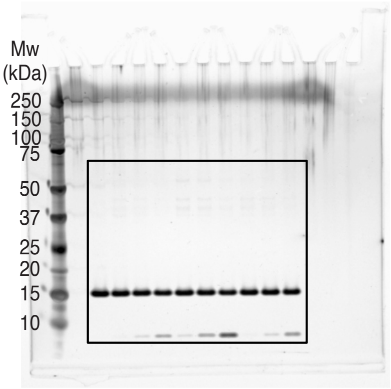

Fig. 1d (*Ub4*)

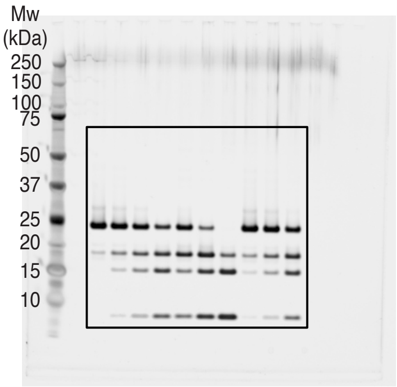

Fig. 1d (*Ub7*)

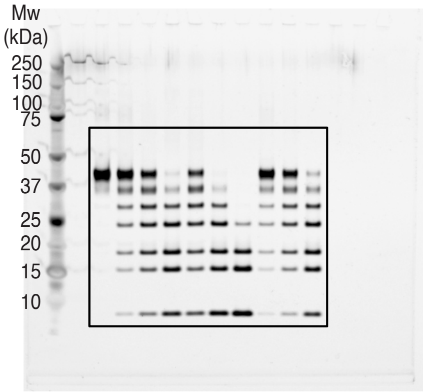

Fig. 1e (*right*)

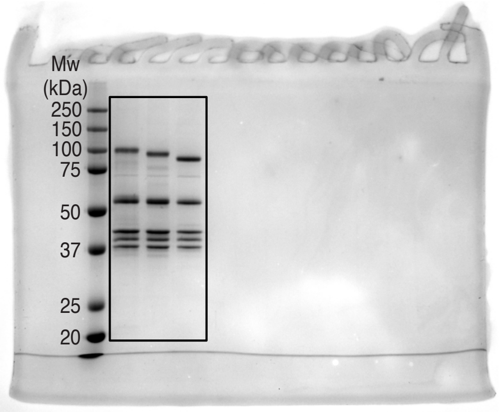

Fig. 1f (*left*)

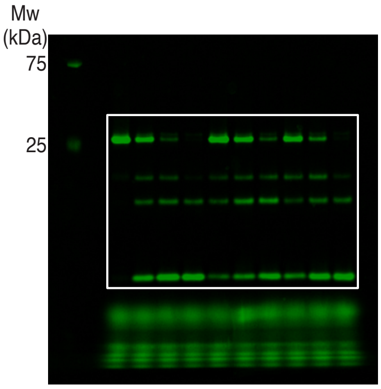

Fig. 1g

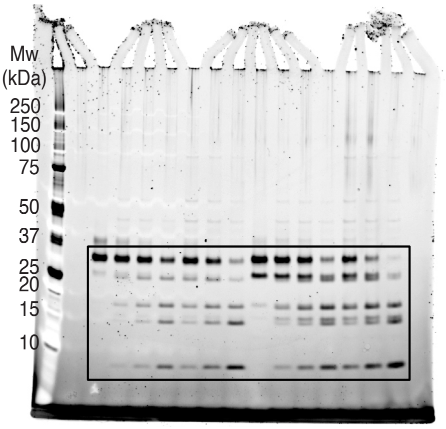

Supplement: Supplementary file 4 [file 41467_2026_75795_MOESM4_ESM.zip › Source Data/Source Data Figure 1.pdf]
