# Supplementary material for: Mechanism of K63-linked polyubiquitin recognition and cleavage by the BRCA1-A complex
Source: Nat Commun. 2026 Aug 11;17:8091. doi: 10.1038/s41467-026-75795-y (PMC13462971; doi:10.1038/s41467-026-75795-y)

Source Data Supplementary Fig. 1

Supplementary Fig. 1a (*left*)

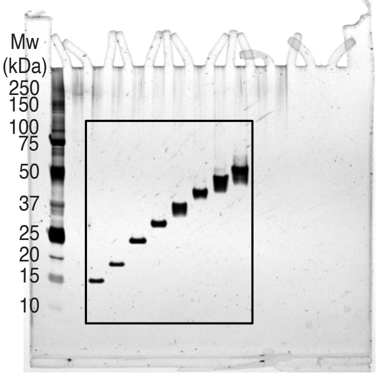

Supplementary Fig. 1b (*left*)

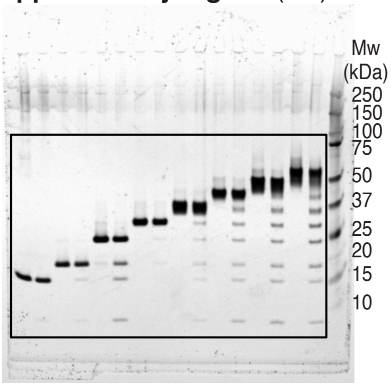

Supplementary Fig. 1b (*right*)

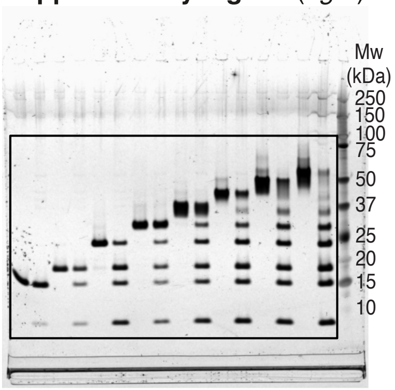

Supplementary Fig. 1a (*right*)

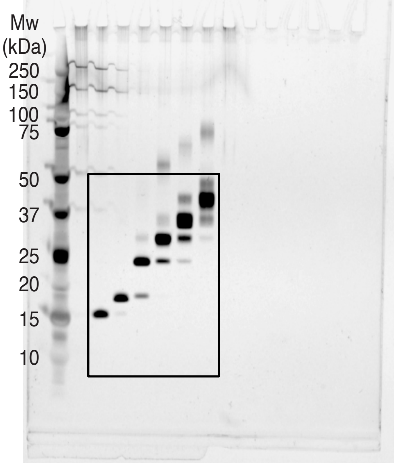

Supplementary Fig. 1c (*Ub2 and Ub3*)

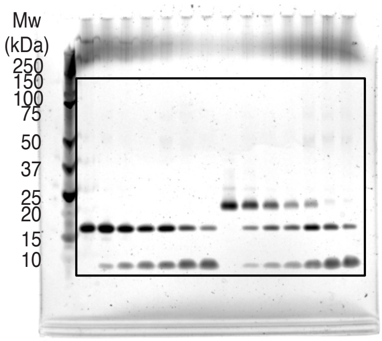

Supplementary Fig. 1c (*Ub4 and Ub5*)

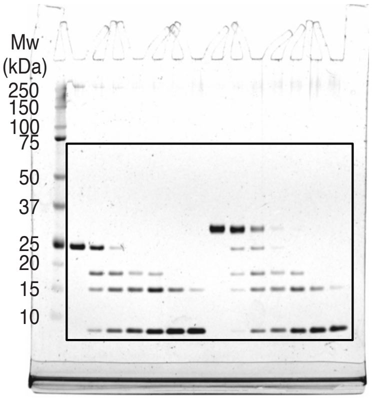

Supplementary Fig. 1c (*Ub6 and Ub7*)

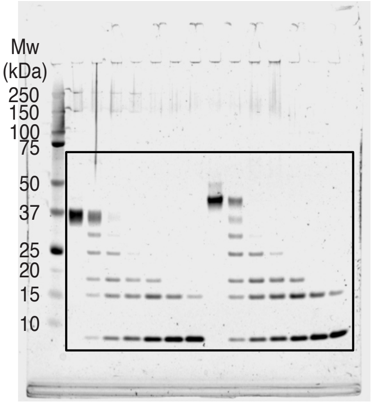

Supplementary Fig. 1c (*Ub8 and Ub9*)

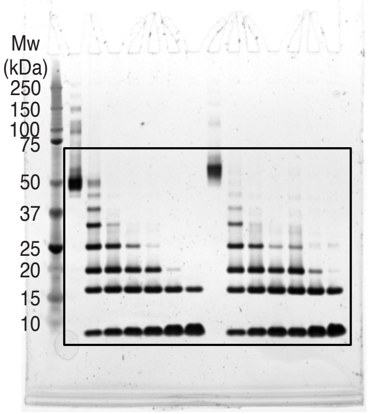

Supplementary Fig. 1d (*top*)

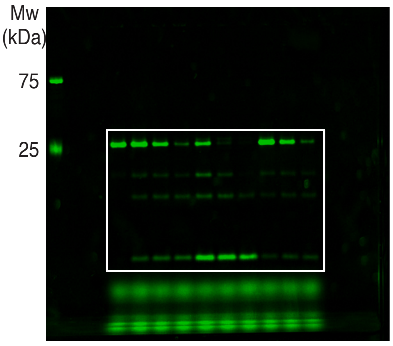

Supplementary Fig. 1e (*top*)

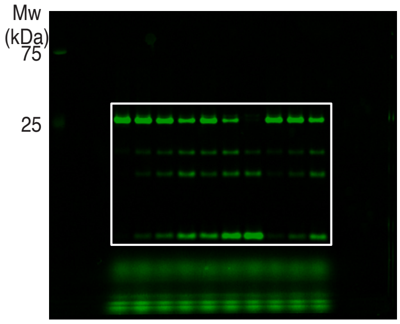

Supplementary Fig. 1f

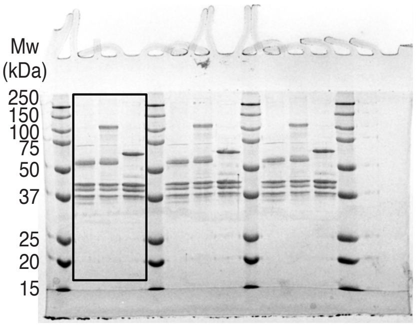

Supplementary Fig. 1g (*Ub2*)

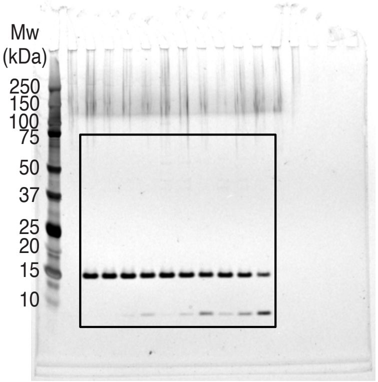

Supplementary Fig. 1g (*Ub4*)

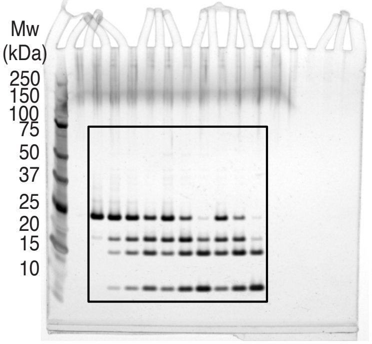

Supplementary Fig. 1g (*Ub7*)

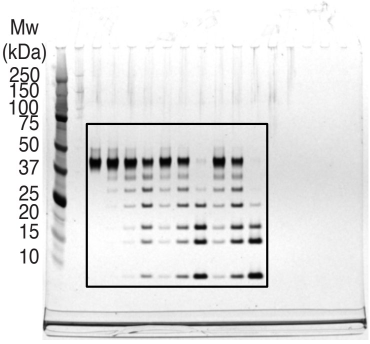

Supplement: Supplementary file 4 [file 41467_2026_75795_MOESM4_ESM.zip › Source Data/Source Data Supplementary Figure 1.pdf]
