# Supplementary material for: Mechanism of K63-linked polyubiquitin recognition and cleavage by the BRCA1-A complex
Source: Nat Commun. 2026 Aug 11;17:8091. doi: 10.1038/s41467-026-75795-y (PMC13462971; doi:10.1038/s41467-026-75795-y)
Supplement: Supplementary file 4 [file 41467_2026_75795_MOESM4_ESM.zip › Source Data/Source Data Supplementary Figure 14.pdf]

Source Data Supplementary Fig. 14

Supplementary Fig. 14f (*left*)

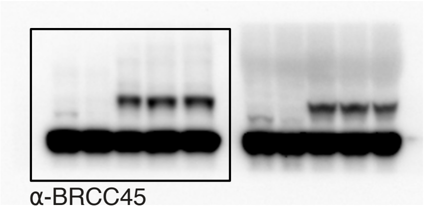
α-BRCC45
α-GAPDH
